# Endothelial Cells Mediated by UCP2 Control the Neurogenic‐to‐Astrogenic Neural Stem Cells Fate Switch During Brain Development

**DOI:** 10.1002/advs.202105208

**Published:** 2022-04-30

**Authors:** Wenwen Wang, Libo Su, Yanyan Wang, Chenxiao Li, Fen Ji, Jianwei Jiao

**Affiliations:** ^1^ State Key Laboratory of Stem Cell and Reproductive Biology Institute of Zoology Chinese Academy of Sciences Beijing 100101 China; ^2^ School of Life Sciences University of Science and Technology of China Hefei 230026 China; ^3^ University of Chinese Academy of Sciences Beijing 100049 China; ^4^ Co‐Innovation Center of Neuroregeneration Nantong University Nantong 226001 China; ^5^ Beijing Institute for Stem Cell and Regenerative Medicine Institute for Stem Cell and Regeneration Chinese Academy of Sciences Beijing 100101 China

**Keywords:** cortical development, endothelial UCP2, neurogenic‐to‐astrogenic fate switch

## Abstract

During mammalian cortical development, neural stem/progenitor cells (NSCs) gradually alter their characteristics, and the timing of generation of neurons and glial cells is strictly regulated by internal and external factors. However, whether the blood vessels located near NSCs affect the neurogenic‐to‐gliogenic transition remain unknown. Here, it is demonstrated that endothelial uncoupling protein 2 (UCP2) deletion reduces blood vessel diameter and affects the transition timing of neurogenesis and gliogenesis. Deletion of endothelial UCP2 results in a persistent increase in astrocyte production at the postnatal stage. Mechanistically, the endothelial UCP2/ROS/ERK1/2 pathway increases chymase‐1 expression to enhance angiotensin II (AngII) secretion outside the brain endothelium. The endotheliocyte‐driven AngII‐gp130‐JAK‐STAT pathway also regulates gliogenesis initiation. Moreover, endothelial UCP2 knockdown decreases human neural precursor cell (hNPC) differentiation into neurons and accelerates hNPC differentiation into astrocytes. Altogether, this work provides mechanistic insights into how endothelial UCP2 regulates the neurogenic‐to‐gliogenic fate switch in the developing neocortex.

## Graphical abstract

Blood vessels reside in close physical proximity to the neural precursor cell (NPC) domains. Endothelial derived signals on NPCs regulate the timing of the neurogenic‐to‐astrocyte fate switch, resulting in an increase in the number of astrocytes in the developing neocortex. This work provides new insights into the regulatory link between blood vessels and sequential differentiation of NPCs.

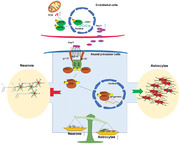

## 1 Introduction

During mammalian neocortical development, neural precursor cells (NPCs) gradually alter their characteristics and morphology to give rise to neurons and glial cells. The timing of neuronal and glial cell generation is strictly regulated.^[^ ^1^, ^2^ ^]^ Biased NPCs produce deep layer neurons first and then upper layer neurons, followed by generating astrocytes at later time points.^[^ ^3^ ^]^ However, the mechanisms that regulate the timing of the neurogenic‐to‐gliogenic fate switch are unclear. One potential explanation for this mechanism is the intrinsic factors such as changes in transcription factor or EGFR receptor expression.^[^ ^4^, ^5^, ^6^ ^]^ The timing of initiation of neurogenesis and the switch to gliogenesis are all altered after loss of the histone methyltransferase enhancer of Zeste homolog 2.^[^ ^7^ ^]^ Another potential explanation for this temporal mechanism is the premature production of astrocytes by NPCs under the influence of external signals. For example, an increase in ciliary neurotrophic factor and neuron‐secreted gliogenic cytokine CT‐1 leads to premature astrocyte formation.^[^ ^8^, ^9^ ^]^ It is worth exploring whether systems other than the nervous system affect the timing of changes in neuronal and glial cells.

At the same time that NPCs give rise to neurons and astrocytes, the central nervous system becomes vascularized.^[^ ^10^ ^]^ Noticeably, blood vessels reside in close physical proximity to the NPC domains.^[^ ^10^, ^11^ ^]^ The cerebrovascular system consisting of highly specific endothelial cells (EC) maintain normal brain function by transporting oxygen and nutrients, regulating the transportation of inflammatory cells and monitoring cellular metabolism.^[^ ^12^, ^13^, ^14^ ^]^ In addition, ECs establish specific vascular niches to maintain normal development, tissue homeostasis, and metabolism by secreting specific vascular factors.^[^ ^15^ ^]^ ECs selectively produce neurotrophin‐3, which maintains adult neural stem cell (NSC) quiescence in the brain and choroid plexus.^[^ ^16^, ^17^ ^]^ However, the role of the developing vascular system in NPCs, particularly in regulating the neurogenic‐to‐gliogenic transition, is largely unexplored.

Mitochondrial uncoupling protein 2 (UCP2) inhibits reactive oxygen species (ROS) production by separating mitochondrial respiration from ATP synthesis in various cell types.^[^ ^18^, ^19^ ^]^ Mitochondria are signal organelles in the endotheliocyte of blood vessels that produce pro‐angiogenic ROS, but not as an energy source because ECs are glycolytic.**^[^** ^20^ **^]^** Whether UCP2 plays a role in EC and affects neurogenic‐to‐gliogenic transition processes remain unknown.

Here, we found that endothelial UCP2 deletion reduces the diameter of blood vessels and affects the transition timing of neurogenesis and gliogenesis. Loss of endothelial UCP2 results in persistently increased astrocyte production during the postnatal stage. Mechanistically, ERK1/2 pathway increases chymase‐1 (CMA1) expression to enhance angiotensin II (AngII) secretion outside the brain endothelium. In addition, endotheliocyte‐driven AngII binds to the neural precursor receptor (angiotensin II type 1a receptor, Agtr1a) to activate the JAK‐STAT pathway, which regulates the initiation of gliogenesis. Moreover, knockdown of endothelial UCP2 enhances the human NPC differentiation into astrocytes and decreases human NPC differentiation into neurons. In this study, we demonstrated that EC‐derived angiotensin signaling is required for the timing of the neurogenic‐to‐astrogenic fate switch during embryonic development.

## 2 Results

### 2.1 The Developing Blood Vessels is Associated with Neural Precursor Cells in the Brain Cortex

**Figure 1:**
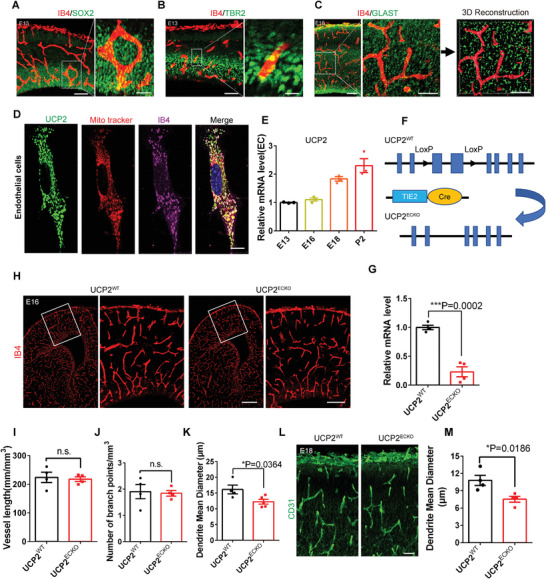
Deletion of endothelial UCP2 reduces the blood vessel diameter. A) Confocal immunofluorescence image of IB4 and SOX2 at E13 showed that blood vessels were located near NPCs. The right is higher magnification images. Scale bars, 100 µm (left), 20 µm (right). B) Confocal immunofluorescence image of IB4 and TBR2 at E13 showed that blood vessels were located near intermediate precursor cells. The right is higher magnification images. Scale bars, 100 µm (left), 20 µm (right). C) Confocal immunofluorescence image of IB4 and GLAST at E16 showed that blood vessels were located near astrocyte precursor cells (APCs). The right is higher magnification images and 3D reconstructions of z‐stacks of brain vessels and APCs. Scale bars, 100 µm (left), 50 µm (right). D) Confocal immunofluorescence image of UCP2, Mito tracker and IB4 in endothelial cells showing the subcellular localization of UCP2 in ECs. Scale bar, 10 µm. E) RT‐PCR was performed to detect the mRNA levels of endothelial UCP2 at E13, E16, E18, and P2 (mean ± SEM, *n* = 3 mice each group). F) Schematic of the endothelial UCP2 conditional knockout mice construction strategy. G) RT‐PCR analysis of the mRNA expression level of UCP2 in the *UCP2^WT^* and *UCP2^ECKO^* isolated brain endothelial cells. ***P < 0.001 (mean ± SEM, *UCP2^WT^ n* = 5 mice; *UCP2^ECKO^ n* = 4 mice). H) Confocal immunofluorescence image of IB4 at E16 in the *UCP2^WT^* and *UCP2^ECKO^* cortical sections. The right is higher magnification images. Scale bars, 200 µm (left), 100 µm (right). I–K) Quantification of the vessel length (I), branch points (J), and mean diameter (K), and showing a decreased mean diameter in *UCP2^ECKO^* cortical sections. *P < 0.05, n.s., not significant (mean ± SEM, unpaired two‐tailed Student's *t* test, I) *n* = 4 mice each group, J) *n* = 4 mice each group, K) *UCP2^WT^ n* = 4 mice; *UCP2^ECKO^ n* = 5 mice). L) Confocal immunofluorescence image of CD31 in the *UCP2^WT^* and *UCP2^ECKO^* cortical sections. Scale bars, 50 µm. M) Quantification of mean diameter showing a decreased mean diameter in *UCP2^ECKO^* cortical sections *P < 0.05 (mean ± SEM, unpaired two‐tailed Student's *t* test, *n* = 4 each group from 3 independent experiments). Data are represented as means ± SEM. unpaired two‐tailed Student's *t* test; At least three biological replicates are shown. n.s., not significant, *P < 0.05, **P < 0.01, ***P < 0.001.

During brain development, blood vessels residing in close physical proximity to the NPC domains act as important components of the neurogenic niche.^[^ ^10^, ^21^ ^]^ We first assessed the positional relationship between NPCs and blood vessels at embryonic day (E) 13. The results revealed that blood vessels, as visualized using biotinylated isolectin B4 (IB4), were located near NPCs (SOX2) and intermediate progenitor cells (TBR2) (**Figure** **1**A,B). We also found that blood vessels resided in close physical proximity to glial progenitor cells (GLAST) at E16 (Figure 1C; Figure S1A, Supporting Information). This finding suggests that blood vessels play a role in NPCs, gradually giving rise to neurons and astrocytes. Previous studies have demonstrated that UCP2 protects endothelial function in diet‐induced obese mice and that UCP2 deficiency leads to premature senescence and mitochondrial fragmentation.^[^ ^22^, ^23^ ^]^ To investigate the role of UCP2 in the developing vascular and nervous system, we first examined the expression of UCP2 in ECs, NPCs, neurons, and astrocytes. The results indicated that UCP2 expression in ECs was the highest compared to that in other cells (Figure S1B, Supporting Information). Next, subcellular localization of UCP2 in ECs was detected by cellular immunostaining. We found that UCP2 was colabeled with MitoTracker in primary ECs (Figure 1D). Real‐time PCR (RT‐PCR) also showed that UCP2 gradually increased from E13 to postnatal day (P) 2 in ECs (Figure 1E), revealing a temporal correlation between NPCs giving rise to neurons and astrocytes and *UCP2* expression in ECs during embryonic development. This finding prompted us to ask whether UCP2 in the developing vascular system affects the temporal changes in neural precursor cells.

### 2.2 Loss of Endothelial UCP2 Reduces the Diameter of the Blood Vessel

To investigate the function of endothelial UCP2 in cortex development, we generated endothelial conditional knockout mice (*UCP2^ECKO^*) by crossing *UCP2* floxed mice (*UCP2^fl/fl^*) with a Tie2‐Cre**^[^** ^24^ **^]^** driver that targets the endothelial cell lineage (Figure 1F). We first used UCP2 and IB4 double cell immunofluorescent staining to determine the EC specificity of Tie2‐Cre‐mediated ablation in the perinatal mouse brain. The results showed that the expression of UCP2 was depleted in *UCP2^ECKO^* brain ECs (Figure S1C,D, Supporting Information). Furthermore, RT‐PCR and western blot analysis showed that UCP2 expression was decreased in *UCP2^ECKO^* isolated brain ECs at E18.5, compared to that in *UCP2^WT^* (Figure 1G; Figure S1E, Supporting Information). Next, we examined whether cerebral vascular morphology changed after UCP2 knockout in ECs. IB4 immunostaining results showed that vessel length and number of branch points did not change after deletion of endothelial UCP2, but the average diameter of the blood vessels decreased compared to that of *UCP2^WT^* littermates at E16 (Figure 1H–K). In addition, immunostaining for another blood vessel marker, CD31, revealed similar results at E18 (Figure 1L,M). To further investigate whether UCP2 deletion has a lasting effect on vascular structure, we tested the vascular network in the brain cortex at P2. IB4 immunostaining showed persistent disruption of the vascular structure (Figure S1F–I, Supporting Information). These results suggest that the loss of endothelial UCP2 affects the average diameter of the brain vasculature.

To further examine whether endothelial UCP2 regulates pericyte recruitment, we performed PDGFR*β* and IB4 immunofluorescent staining. The results showed that pericyte recruitment to blood vessels was normal in the *UCP2^ECKO^* brain (Figure S1J, Supporting Information). To explore the role of endothelial UCP2 during blood brain barrier (BBB) development, we first evaluated the integrity of endothelial tight junctions in *UCP2^ECKO^* mice. BBB permeability is dependent on microvascular endothelial tight junctions.^[^ ^25^, ^26^ ^]^ We observed that tight junction proteins, claudin‐5 and ZO‐1 had comparable levels between *UCP2^WT^* and *UCP2^ECKO^* mice (Figure S2A–D, Supporting Information). Next, we detected BBB integrity using the fluorescent tracer AlexaFluor 555 cadaverine (Cad‐A555) and found that the integrity of the BBB in *UCP2^ECKO^* mice was consistent with that in *UCP2^WT^* mice (Figure S2E, Supporting Information). The vascular basement membrane provides structural support to the blood vessels and maintains vascular homeostasis.^[^ ^27^ ^]^ Immunostaining for collagen IV (a marker of the EC basement membrane) showed that there was no significant difference between *UCP2^WT^* and *UCP2^ECKO^* mice (Figure S2F,G, Supporting Information). These results suggest that the loss of endothelial UCP2 does not affect BBB integrity.

### 2.3 Loss of Endothelial UCP2 Regulates the Transition Timing of Neurogenesis and Gliogenesis

Blood vessel growth occurs simultaneously as neural progenitor cells sequentially give rise to neurons and astrocytes within the brain.^[^ ^28^ ^]^ To determine whether vascular changes affect nervous system development, we first assessed the self‐renewal ability of NPCs and dividing NPCs. Immunofluorescence staining analysis showed that the number of SOX2^+^ precursor cells was comparable between *UCP2^WT^* and *UCP2^ECKO^* mice at E13 and E16 (Figure S3A–D, Supporting Information), indicating that the deletion of endothelial UCP2 did not affect the self‐renewal of NPCs. In addition, we observed that *UCP2^ECKO^* did not differ significantly in the number of pH3^+^ cells (a marker of dividing cells in the M phase) compared with *UCP2^WT^* in E13 mouse cortices (Figure S3E,F, Supporting Information), indicating that mitotic activity was not affected when endothelial UCP2 was lost.

**Figure 2:**
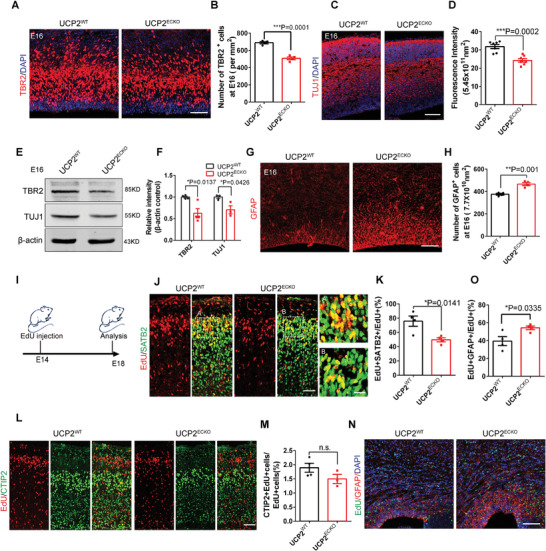
Endothelial UCP2 deletion affects the transition timing of neurogenesis and gliogenesis. A) Confocal immunofluorescence image of TBR2^+^ cells in E16 *UCP2^WT^* and *UCP2^ECKO^* mice. Scale bar, 100 µm. B) Quantification showing the decreased number of TBR2^+^cells in *UCP2^ECKO^* mice. ***P < 0.001 (mean ± SEM, unpaired two‐tailed Student's *t* test, *n* = 4 mice each group). C) Confocal immunofluorescence image of TUJ1^+^ cells in E16 *UCP2^WT^* and *UCP2^ECKO^* mice. Scale bar, 100 µm. D) Quantification showing the decreased fluorescence intensity of TUJ1^+^ cells. ***P < 0.001 (mean ± SEM, unpaired two‐tailed Student's *t* test, *n* = 7 each group from 3 independent experiments). E) Western blot analysis of the expression levels of IPCs marker TBR2 and neurons marker TUJ1. *β*‐actin was detected as loading control. F) Statistics of relative intensity of TBR2 and TBR1 showing the decreased expression of TBR2^+^ and TUJ1^+^cells in *UCP2^ECKO^* mice. *P < 0.05 (mean ± SEM, unpaired two‐tailed Student's *t* test, TBR2 *n* = 4 mice each group; TUJ1 *n* = 3 mice each group). G) Confocal immunofluorescence image of GFAP^+^ cells in E16 *UCP2^WT^* and *UCP2^ECKO^* mice. Scale bar, 100 µm. H) Quantification of the number of GFAP^+^ cells showing the increased GFAP^+^ cells in *UCP2^ECKO^* mice. **P < 0.01 (mean ± SEM, unpaired two‐tailed Student's *t* test, *n* = 4 mice each group). I) Schematic of EdU intraperitoneally to E14 and the analysis of the neurons and astrocytes markers at E18.5. J) Confocal immunofluorescence image of EdU^+^ SATB2^+^ cells in E18 *UCP2^WT^* and *UCP2^ECKO^* mice. The right is magnification images. Scale bars, 50 µm (left), 20 µm (right). K) Quantification of the percentage of EdU^+^SATB2^+^ cells showing decreased EdU^+^SATB2^+^ cells in *UCP2^ECKO^* mice. *P < 0.05 (mean ± SEM, unpaired two‐tailed Student's *t* test, *n* = 4 mice each group). L) Confocal immunofluorescence image of EdU^+^ CTIP2^+^ cells in E18 *UCP2^WT^* and *UCP2^ECKO^* mice. Scale bar, 50 µm. M) Quantification of the percent of EdU^+^CTIP2^+^ cells showing no significant difference in *UCP2^ECKO^* mice. n.s., not significant. (mean ± SEM, unpaired two‐tailed Student's *t* test, *n* = 4 mice each group). N) Confocal immunofluorescence image of EdU^+^ GFAP^+^ cells in E18 *UCP2^WT^* and *UCP2^ECKO^* mice. Scale bar, 100 µm. O) Quantification of the percent of EdU^+^ GFAP ^+^ cells showing increased EdU^+^ GFAP^+^ cells in *UCP2^ECKO^* mice. *P < 0.05 (mean ± SEM, unpaired two‐tailed Student's *t* test, *n* = 4 mice each group). Data are represented as means ± SEM. unpaired two‐tailed Student's *t* test; At least three biological replicates are shown. *P < 0.05, **P < 0.01, ***P < 0.001.

Immunofluorescence staining confirmed that the number of TBR2^+^ intermediate precursor cells and TUJ1^+^ neurons was decreased in *UCP2^ECKO^* mice at E16 (**Figure** **2**A–D). Consistently, western blot analysis confirmed that the expression of TBR2 and TUJ1 were decreased when endothelial UCP2 was absent (Figure 2E,F). We also observed that the number of BLBP^+^ astrocyte progenitors and GFAP^+^ astrocytes increased in *UCP2^ECKO^* mice at E16 (Figure 2G,H; Figure S3G,H, Supporting Information). We then quantified the protein levels of astrocyte progenitor‐related markers, such as BLBP and GLAST, and found that their expression was increased in *UCP2^ECKO^* mice (Figure S3I,J, Supporting Information).

We also addressed whether the loss of endothelial UCP2 regulates the transition timing of neurogenesis/gliogenesis. We performed birth‐date analysis using EdU intraperitoneally in pregnant 15 d mice (E14) and examined the neuronal and astrocyte markers at E18.5 (Figure 2I). The results showed that EdU^+^SATB2^+^ cells were reduced in *UCP2^ECKO^* mice (Figure 2J,K) and that EdU^+^CTIP2^+^ cells had comparable levels between *UCP2^WT^* and *UCP2^ECKO^* mouse brains (Figure 2L,M). Simultaneously, we observed that EdU^+^GFAP^+^ cells were increased in *UCP2^ECKO^* mice compared to those in *UCP2^WT^* brains (Figure 2N,O). Furthermore, we observed that the number of NEUN^+^ cells was decreased and the number of GFAP^+^ cells was increased in *UCP2^ECKO^* mice at E18 (Figure S4A–C, Supporting Information).

We also analyzed the generation of oligodendrocyte lineage cells. Immunostaining analysis showed that the number of Olig2^+^ cells (a marker for oligodendrocytes) was not significantly different between *UCP2^WT^* and *UCP2^ECKO^* (Figure S4D,E, Supporting Information), indicating that loss of endothelial UCP2 did not affect the generation of oligodendrocytes. These results indicate that the deletion of endothelial UCP2 accelerated the switch from superficial layer neurogenesis to gliogenesis.

To determine whether the overall development of the cortex was affected in endothelial UCP2 knockout mice, we investigated neuronal development at P0. Immunostaining analysis showed that the number of SATB2^+^ and CTIP2^+^ neurons was decreased in the brains of *UCP2^ECKO^* mice (Figure S4F–H, Supporting Information). In addition, western blot analysis confirmed that the expression of NEUN and TUJ1 was decreased when endothelial UCP2 was absent (Figure S4I,J, Supporting Information). We have showed some western blots on key proteins responsible for synaptic integrity and function, such as p‐CREB, synaptophysin in brain cortical tissue at P2 and P8. The results showed that synaptic integrity and function had no effect after the deletion of endothelial UCP2 (Figure S4K–N, Supporting Information).

To analyze the effects of apoptosis in mice, we performed a terminal deoxynucleotidyl transferase‐mediated dUTP end‐labeling assay to assess cells apoptosis. The results showed that there was no significant difference between *UCP2^WT^* and *UCP2^ECKO^* cortices, suggesting that the reduction of neurons in the *UCP2^ECKO^* cortex was not caused by apoptosis (Figure S5A,B, Supporting Information).

### 2.4 Deletion of Endothelial UCP2 Leads to Persistent Increase of Astrocyte Production

**Figure 3:**
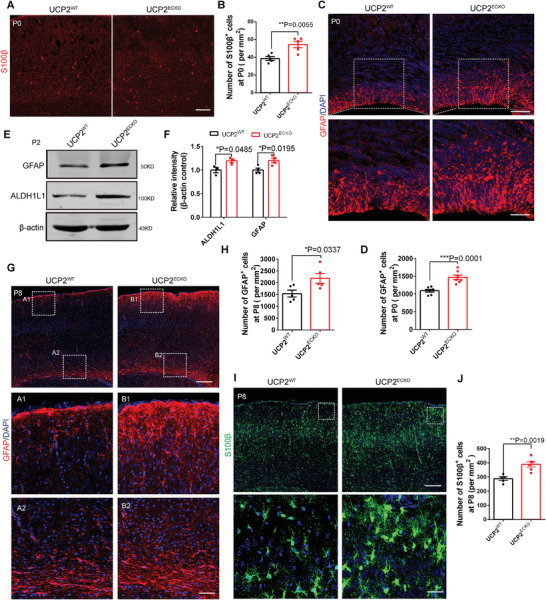
Loss of endothelial UCP2 leads to persistent increased astrocyte production. A) Confocal immunofluorescence image of S100*β* ^+^ cells in P0 *UCP2^WT^* and *UCP2^ECKO^* mice. Scale bar, 100 µm. B) Quantification of the number of S100*β* ^+^ cells showing increased S100*β* ^+^ cells in *UCP2^ECKO^* mice. **P < 0.01 (mean ± SEM, unpaired two‐tailed Student's *t* test, *n* = 5 mice each group). C) Confocal immunofluorescence image of GFAP^+^ cells in P0 *UCP2^WT^* and *UCP2^ECKO^* mice. The down is higher magnification images. Scale bars, 100 µm (up), 50 µm (down). D) Quantification of the number of GFAP^+^ cells showing increased GFAP^+^ cells in *UCP2^ECKO^* mice. ***P < 0.001 (mean ± SEM, unpaired two‐tailed Student's *t* test, *n* = 7 each group from 3 independent experiments). E) Western blot analysis of the expression levels of astrocyte marker GFAP and ALDH1L1. *β*‐actin was detected as loading control. F) Statistics of relative intensity of GFAP and ALDH1L1 showing increased the expression of GFAP^+^ and ALDH1L1^+^ cells in P2 *UCP2^ECKO^* mice. *P < 0.05 (mean ± SEM, unpaired two‐tailed Student's *t* test, GFAP *n* = 3 mice each group; ALDH1L1 *n* = 4 mice each group). G) Confocal immunofluorescence image of GFAP^+^ cells in P8 *UCP2^WT^* and *UCP2^ECKO^* mice. A1, A2, B1, and B2 are higher magnification images. Scale bars, 200 µm (up), 50 µm (down). H) Quantification of the number of GFAP^+^ cells showing increased GFAP^+^ cells in *UCP2^ECKO^* mice. *P < 0.05 (mean ± SEM, unpaired two‐tailed Student's *t* test, *n* = 5 each group from 3 independent experiments). I) Confocal immunofluorescence image of S100*β* ^+^ cells in P8 *UCP2^WT^* and *UCP2^ECKO^* mice. The down is higher magnification images. Scale bars, 200 µm (up), 50 µm (down). J) Quantification of the number of S100*β* ^+^ cells showing increased S100*β* ^+^ cells in *UCP2^ECKO^* mice. **P < 0.01 (mean ± SEM, unpaired two‐tailed Student's *t* test, UCP2^WT^ *n* = 5 each group, UCP2^ECKO^ *n* = 6 each group from 3 independent experiments). Data are represented as means ± SEM. unpaired two‐tailed Student's *t* test; At least three biological replicates are shown. *P < 0.05. **P < 0.01, ***P < 0.001.

To test whether astrogenesis was accelerated in the neocortical regions at the postnatal stage when endothelial UCP2 was absent, we performed a series of immunostaining and western blot analyses to detect changes in astrogenesis at different postnatal stages. We observed that the number of S100*β* ^+^ and GFAP^+^ astrocytes in *UCP2^ECKO^* mice was obviously higher than that in wild‐type mice at P0 (**Figure** **3**A–D). In addition, western blot analysis showed that the expression of the astrocyte markers GFAP and ALDH1L1 was increased in *UCP2^ECKO^* mouse brains at P2 (Figure 3E,F). Furthermore, we observed that the number of GFAP^+^ and S100*β* ^+^ astrocytes was increased in *UCP2^ECKO^* mice at P8 (Figure 3G–J). These results suggest that the production of astrocytes persistently increased in the cerebral cortex of *UCP2^ECKO^* mice.

Astrocytes and microglia are the main cells responsible for the inflammatory responses in the brain.^[^ ^29^ ^]^ To determine whether the persistent increase in astrocytes is caused by an inflammatory response, we examined whether endothelial UCP2 deletion affects microglia and inflammation. Immunostaining results showed that the number of IBA1^+^ and CD68^+^ (microglia marker) cells was not significantly different between *UCP2^WT^* and *UCP2^ECKO^* mice (Figure S5C–E, Supporting Information), suggesting that endothelial UCP2 deletion had no effect on microglial cells. Furthermore, we measured the protein levels of the pro‐inflammatory cytokines IL‐6 and IL‐1*β* in the brain and found that their expression was not significantly changed after endothelial UCP2 deletion (Figure S5F,G, Supporting Information). These results suggest that there was no inflammation in *UCP2^ECKO^* mice and that the persistent increase in astrocytes in *UCP2^ECKO^* mice is not caused by an inflammatory response.

### 2.5 ERK1/2 Signaling Upregulates CMA1 to Increase AngII in UCP2 Knockout ECs

**Figure 4:**
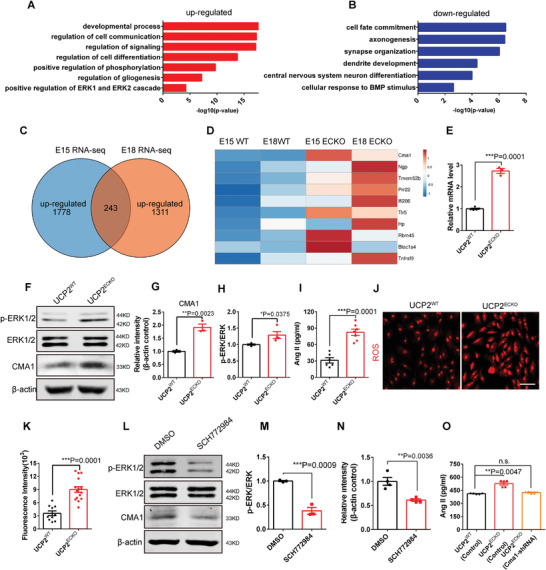
ERK1/2 signaling upregulates CMA1 to increase Ang II in UCP2 knockout endothelial cells. A,B) Gene Ontology (GO) analysis of biological processes related to the upregulated and downregulated genes in the UCP2 knockout endothelial cells. C) Schematic diagram of the common up‐regulated genes from RNA‐Seq of E15 and E18. D) Heat map of 10 differentially expressed genes from RNA‐Seq of E15 and E18. E) RT‐PCR analysis of the mRNA expression level of CMA1 in the *UCP2^WT^* and *UCP2^ECKO^* isolated brain endothelial cells. ***P < 0.001 (mean ± SEM, unpaired two‐tailed Student's *t* test, *UCP2^WT^ n* = 5mice; *UCP2^ECKO^ n* = 3 mice). F) Western blot analysis of the expression levels of CMA1, p‐ERK1/2, and ERK1/2. *β*‐actin was detected as loading control. G) Statistics of relative intensity of CMA1 showing the increased expression of CMA1 in *UCP2^ECKO^* isolated brain endothelial cells. **P < 0.01 (mean ± SEM, unpaired two‐tailed Student's *t* test, *n* = 3 mice). H) Statistics showing the increased ratio of p‐ERK1/2 and ERK1/2 in *UCP2^ECKO^* isolated brain endothelial cells. *P < 0.05 (mean ± SEM, unpaired two‐tailed Student's *t* test, *UCP2^WT^ n* = 3 mice; *UCP2^ECKO^ n* = 4 mice). I) ELISA analysis by collecting supernatant in isolated *UCP2^WT^* and *UCP2^ECKO^* mice brain ECs showing increased Ang II in endothelial UCP2 knockout mice. ***P < 0.001 (mean ± SEM, unpaired two‐tailed Student's *t* test, *UCP2^WT^ n* = 6; *UCP2^ECKO^ n* = 7 from 3 independent experiments). J) Confocal immunofluorescence image of ROS level labeled by DHE in *UCP2^WT^* and *UCP2^ECKO^* isolated brain primary endothelial cells. Scale bar, 100 µm. K) Quantification showing the increased ROS level in *UCP2^ECKO^* isolated brain primary endothelial cells. ***P < 0.001(mean ± SEM, unpaired two‐tailed Student's *t* test, *UCP2^WT^ n* = 10; *UCP2^ECKO^ n* = 15 from 3 independent experiments). L) Western blot analysis of the expression levels of CMA1, p‐ERK1/2 and ERK1/2 in bEnd.3 treated with the inhibitor of ERK1/2 SCH772984. *β*‐actin was detected as loading control. M,N) Statistics showing reduced the ratio of p‐ERK1/2 and ERK1/2 (M) and CMA1 (N) in bEnd.3 treated with SCH772984 compared with control. ***P < 0.001(mean ± SEM, unpaired two‐tailed Student's *t* test, *n* = 3 independent experiments (M); *n* = 4 independent experiments (N)). O) ELISA analysis showing that knockdown of CMA1 significantly restored Ang II increase due to endothelial UCP2 knockout. **P < 0.01 (mean ± SEM, one‐way ANOVA with Dunnett's multiple‐comparison correction, *n* = 5 each group from 3 independent experiments). Data are represented as means ± SEM. unpaired two‐tailed Student's *t* test; one‐way ANOVA with Dunnett's multiple‐comparison correction, n.s., not significant, *P < 0.05, **P < 0.01, ***P < 0.001.

Endothelial UCP2 deletion is necessary for the transition timing of neurogenesis and gliogenesis and the persistent increase in astrocyte production during brain development. Therefore, we explored the mechanism of endothelial UCP2 deletion by RNA sequencing (RNA‐seq) brain ECs. Gene ontology analysis of the upregulated genes showed obvious enrichment of biological processes related to the regulation of cell signaling, cell communication, and gliogenesis (**Figure** **4**A). Gene ontology analysis of the downregulated genes showed obvious enrichment of biological processes related to neuronal differentiation (Figure 4B). Moreover, we selected the commonly upregulated genes from the RNA‐Seq of E15 and E18 brain ECs (Figure 4C). Heat maps showed 10 highly differentially expressed genes from RNA‐Seq of E15 and E18 brain ECs (Figure 4D). We noted that the mast cell chymase gene (*CMA1*) was a promising candidate molecule, and RT‐PCR analysis confirmed that CMA1 was significantly upregulated in isolated *UCP2^ECKO^* mice brain ECs (Figure 4E). Western blotting confirmed that the expression of CMA1 was increased, and the expression of phosphorylated ERK1/2 (p‐ERK1/2) was also increased in isolated *UCP2^ECKO^* mouse brain ECs (Figure 4F–H).

CMA1 is important for the formation of AngII.**^[^** ^30^ **^]^** We performed AngII ELISA analysis by collecting supernatants from isolated *UCP2^WT^* and *UCP2^ECKO^* mouse brain ECs. The results showed a significant increase in endothelial UCP2 knockout mice (Figure 4I). To further explore the reasons for the increase in CMA1, we assessed mitochondrial respiration expressed as oxygen consumption rate in primary ECs of *UCP2^WT^* and *UCP2^ECKO^* mice. The results showed that *UCP2^ECKO^* mice were reduced in mitochondrial basal and maximal respiration and ATP production compared to *UCP2^WT^* mice (Figure S6A, Supporting Information). Furthermore, extracellular acidification rate (ECAR) analysis showed that *UCP2^ECKO^* mice were decreased in mitochondrial basal ECAR and glycolytic capacity compared to *UCP2^WT^* mice (Figure S6B, Supporting Information). Previous studies have also demonstrated that UCP2 regulates the production of ROS.^[^ ^31^, ^32^ ^]^ To test this, we used different concentrations of hydrogen peroxide (H_2_O_2_) and genipin (UCP2 selective inhibitor) to treat primary ECs. Dihydroethidium (DHE) staining showed that ROS production tended to increase with increasing concentrations of H_2_O_2_ and genipin (Figure S6C–F, Supporting Information). These results suggested that deletion of endothelial UCP2 affects cellular metabolism. Confocal microscopy images showed that mitochondria length of endothelial cells was decreased in *UCP2^ECKO^* mice (Figure S6G,H, Supporting Information). These data suggest that *UCP2* deletion in endothelial cells led to abnormal mitochondrial morphology.

We further analyzed the brain ECs isolated from *UCP2^WT^* and *UCP2^ECKO^* mice with DHE and found that ROS production was increased in endothelial UCP2 knockout mice (Figure 4J,K). In addition, p‐ERK1/2 was increased in *UCP2^ECKO^* mice isolated from brain ECs (Figure S6I–K, Supporting Information). Western blot analysis confirmed that SCH772984, a novel specific inhibitor of ERK1/2, significantly downregulated p‐ERK1/2 and CMA1 expression in bEnd.3 brain ECs (Figure 4L–N). AngII ELISA analysis also showed a significant increase in primary ECs infected with CMA1 (Figure S6K, Supporting Information). Knockdown of CMA1 significantly restored AngII increase induced by endothelial UCP2 knockout (Figure 4O). These findings indicate that UCP2/ROS/ERK1/2 signaling upregulates CMA1 to increase AngII levels in *UCP2^ECKO^* mouse brain ECs.

### 2.6 Endotheliocyte‐Driven AngII‐gp130‐JAK‐STAT Pathway Regulates the Initiation of Gliogenesis

To evaluate the role of brain endothelial CMA1 in neurogenesis and gliogenesis, we first measured the expression of CMA1 in ECs. Brain slices and cell immunofluorescence staining showed that CMA1 was expressed in IB4 ‐labeled ECs (Figure S7A,B, Supporting Information). Using cocultured Transwell systems, we detected the effect of endothelial CMA1 on NPC fate determination. Immunofluorescence staining showed that endothelial CMA1 overexpression reduced the proportion of neurons and increased the proportion of astrocytes (Figure S7C–E, Supporting Information). Western blot analysis confirmed that the overexpression of endothelial CMA1 reduced the expression of TUJ1^+^ neurons and increased the expression of GFAP^+^ astrocytes (Figure S7F,G, Supporting Information). Interestingly, we also found that the expression level of pSTAT3 was increased, suggesting the role of STAT3 signaling pathways in initiating gliogenesis (Figure S7F,H, Supporting Information).

**Figure 5:**
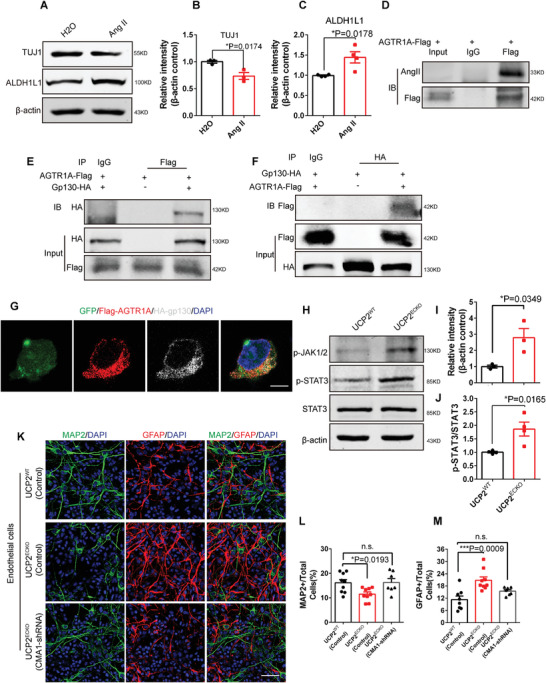
Endotheliocyte‐driven AngII‐gp130‐JAK‐STAT pathway regulates initiation of gliogenesis. A) Western blot analysis of the expression levels of TUJ1, GFAP, STAT3, and p‐STAT3 in primary NPCs treated with Ang II. *β*‐actin was detected as loading control. B) Statistics of the relative intensity of TUJ1 showing the decreased expression of TUJ1 in primary NPCs treated with Ang II. *P < 0.05 (mean ± SEM, unpaired two‐tailed Student's *t* test, *n* = 3 independent experiments). C) Statistics of relative intensity of ALDH1L1 showing the increased expression of ALDH1L1 in primary NPCs treated with Ang II. *P < 0.05 (mean ± SEM, unpaired two‐tailed Student's *t* test, *n* = 4 independent experiments). D) Immunoprecipitation (IP) experiment showing the interaction between Ang II and AGTR1A. E,F) Co‐immunoprecipitation (CO‐IP) assays for the interaction between AGTR1A and gp130. G) Confocal immunofluorescence image of FLAG and HA showing the colocalization of AGTR1A and gp130. Scale bar, 5 µm. H) Western blot analysis of the expression levels of p‐JAK1/2, STAT3, and p‐STAT3 in UCP2WT and UCP2ECKO cerebral cortex. *β*‐actin was detected as loading control. I) Statistics showing the increased expression levels of p‐JAK1/2 in *UCP2^ECKO^* cerebral cortex. *P < 0.05 (mean ± SEM, unpaired two‐tailed Student's *t* test, *n* = 3 mice each group). J) Statistics showing the increased ratio of p‐STAT3 and STAT3 in *UCP2^ECKO^* cerebral cortex. *P < 0.05 (mean ± SEM, unpaired two‐tailed Student's *t* test, *n* = 4 mice each group). K) Confocal immunofluorescence image showed that endothelial CMA1 knockdown can rescue the developmental imbalance caused by endothelial *UCP2* deletion. Scale bar, 50 µm. L,M) Quantification of the percent of MAP2^+^ cells and GFAP^+^ cells. *P < 0.05, ***P < 0.001 (mean ± SEM, one‐way ANOVA with Dunnett's multiple‐comparison correction, *n* = 5 each group from 3 independent experiments). Data are represented as mean ± SEM. unpaired two‐tailed Student's *t* test; one‐way ANOVA with Dunnett's multiple‐comparison correction, *P < 0.05, ***P < 0.001.

To analyze the role of brain endothelial AngII in neurogenesis and gliogenesis, we cultured primary NPCs stimulated with AngII. Immunofluorescence staining showed that exogenous AngII reduced the proportion of neurons and increased the proportion of astrocytes (Figure S7I–K, Supporting Information). Western blot analysis confirmed that exogenous AngII reduced the number of TUJ1^+^ neurons and increased the number of ALDH1L1^+^ astrocytes (**Figure** **5**A–C). Then, we explored how endothelial AngII triggers gliogenesis. Previous studies have reported that Agtr1a is a receptor for AngII.^[^ ^33^, ^34^ ^]^ Thus, we performed an immunoprecipitation experiment to test the interaction between AngII and AGTR1A. The results showed that Flag‐tagged AGTR1A pulled down AngII (Figure 5D), suggesting that the effect of AngII was mediated by AGTR1A.

We also tested whether AGTR1A forms a signaling complex with other signal‐transducing receptors, such as glycoprotein 130 (gp130), which dimerizes and initiates intracellular signaling.^[^ ^35^, ^36^ ^]^ Co‐immunoprecipitation assays were performed to test the interaction between AGTR1A and gp130. The results showed that Flag‐tagged AGTR1A pulled down HA‐tagged gp130 and that HA‐tagged gp130 similarly pulled down Flag‐tagged AGTR1A (Figure 5E,F), suggesting that there was a direct interaction between AGTR1A and gp130. To visualize their interaction, we performed immunofluorescence staining after cotransfection with Flag‐AGTR1A and HA‐gp130 plasmids. The results showed that HA‐gp130 colocalized with Flag‐ AGTR1A in the cell membrane of N2a cells (Figure 5G).

Finally, we also found that the expression levels of p‐JAK1/2 and p‐STAT3 were increased in endothelial UCP2 knockout mice (Figure 5H–J). Together, the endotheliocyte‐driven AngII‐gp130‐JAK‐STAT pathway regulates the initiation of gliogenesis. Using cocultured Transwell systems, we determined whether endothelial CMA1 knockdown could rescue the developmental imbalance caused by endothelial *UCP2* deletion. We found that CMA1‐shRNA rescued the decrease in the number of neurons and the increase in astrocytes in endothelial UCP2 knockout mice brain ECs (Figure 5K–M). In summary, these results suggest that the loss of endothelial UCP2 regulates the transition timing of neurogenesis and gliogenesis and results in persistent increase in astrocyte production by increasing endotheliocyte‐driven AngII to activate the JAK‐STAT pathway during brain development (Figure S8D, Supporting Information).

### 2.7 Knockdown of Endothelial UCP2 Accelerates the Differentiation of Human Neural Precursor Cells Toward Astrocytes

**Figure 6:**
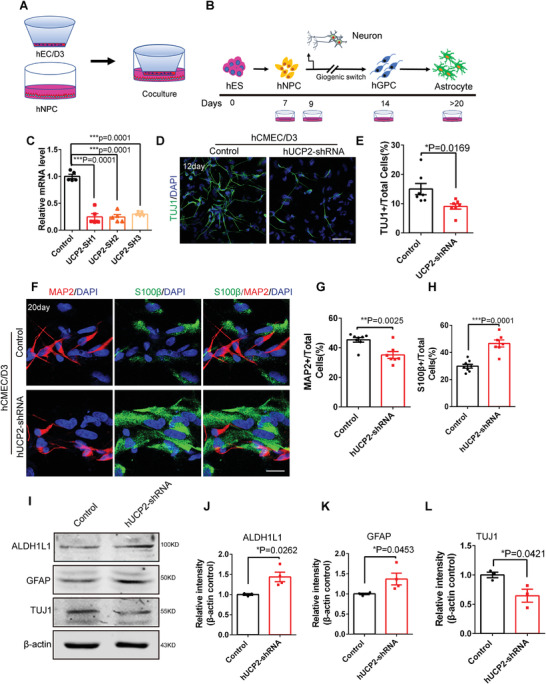
Knockdown of endothelial UCP2 accelerates human NPCs differentiation toward astrocytes. A) The diagram of human CMEC/D3 and human NPCs coculture system. B) Schematic of human embryonic stem cells differentiate into human NPCs, followed by neurons and astrocytes. C) RT‐PCR analysis showing the knockdown efficiency of human UCP2‐shRNA at the mRNA level. ***P < 0.001(mean ± SEM, one‐way ANOVA with Dunnett's multiple‐comparison correction, *n* = 5 each group from 3 independent experiments). D) Confocal immunofluorescence image of TUJ1^+^neurons at 12 days of differentiation from human ESCs. Scale bar, 100 µm. E) Quantification of the percent of TUJ1^+^ cells showing decreased TUJ1^+^cells in human NPCs cocultured with UCP2‐shRNA lentivirus‐infected with hCMEC/D3 cells line. *P < 0.05 (mean ± SEM, unpaired two‐tailed Student's *t* test, *n* = 7 each group from 3 independent experiments). F) Confocal immunofluorescence image of MAP2^+^neurons and S100*β* ^+^ astrocytes at 20 d of differentiation from human ESCs. Scale bars, 20 µm. G,H) Quantification of the percent of MAP2^+^ neurons and S100*β* ^+^ astrocytes showing decreased MAP2^+^ neurons and increased S100*β*+ astrocytes in human NPCs cocultured with UCP2‐shRNA lentivirus‐infected with hCMEC/D3 cells line. **P < 0.001, ***P < 0.0001 (mean ± SEM, unpaired two‐tailed Student's *t* test, Control *n* = 8; UCP2‐shRNA *n* = 7 from 3 independent experiments). I) Western blot analysis of the expression levels of ALDH1L1, GFAP, TUJ1 in human NPCs cocultured with UCP2‐shRNA lentivirus‐infected with hCMEC/D3 cells line. *β*‐actin was detected as loading control. J,K) Statistics of relative intensity of ALDH1L1 and GFAP showing the increased expression of ALDH1L1 and GFAP in human NPCs cocultured with UCP2‐shRNA lentivirus‐infected with hCMEC/D3 cells line. *P < 0.05 (mean ± SEM, unpaired two‐tailed Student's *t* test, ALDH1L1: Control *n* = 3; UCP2‐shRNA *n* = 4; GFAP: *n* = 4 each group from 3 independent experiments). L) Statistics of relative intensity of TUJ1 showing the decreased expression of TUJ1 in human NPCs cocultured with UCP2‐shRNA lentivirus‐infected with hCMEC/D3 cells line. *P < 0.05 (mean ± SEM, unpaired two‐tailed Student's *t* test, *n* = 3 independent experiments). Data are represented as means ± SEM. unpaired two‐tailed Student's *t* test; *P < 0.05, ***P < 0.001.

To examine whether endothelial UCP2 has a similar function in human NPCs, we performed human CMEC/D3 and human NPC coculture Transwell experiments. Human NPCs were formed after 7 d of differentiation from H9 human embryonic stem cells (ESCs) and then cocultured with human CMEC/D3 cells infected with UCP2‐shRNA and control (**Figure** **6**A,B). We first constructed a human UCP2 knockdown plasmid and confirmed the knockdown efficiency of human UCP2‐shRNA at the mRNA level (Figure 6C). Compared with the control, UCP2‐shRNA lentivirus‐infected hCMEC/D3 cells reduced the proportion of TUJ1^+^ neurons after 12 d of differentiation from human ESCs (Figure 6D,E). The UCP2‐shRNA lentivirus‐infected hCMEC/D3 cell line reduced the proportion of MAP2^+^ neurons and increased the proportion of S100*β* ^+^ astrocytes compared with the control at 20 d of differentiation from human ESCs (Figure 6F–H). Western blot analysis further confirmed that UCP2‐shRNA lentivirus‐infected hCMEC/D3 cells had reduced expression of TUJ1^+^ neurons and increased expression of GFAP^+^ and ALDH1L1^+^ astrocytes (Figure 6I–L). These results suggest that endothelial UCP2 plays an essential role in the differentiation of human NPCs. Interestingly, we performed an experiment on hNPCs stimulated with AngII and found that exogenous AngII reduced the proportion of MAP2^+^ neurons and increased the proportion of GFAP^+^ astrocytes (Figure S8A–C, Supporting Information). These results demonstrate that AngII enhances human NPC differentiation into astrocytes and decreases human NPC differentiation into neurons.

## 3 Discussion

A fundamental question in understanding brain development is how neural progenitor cells and NSCs produce various cell types to achieve an appropriate brain structure. The timing of the neurogenic‐to‐astrogenic fate switch in NPCs is strictly regulated.^[^ ^37^ ^]^ In this study, we have shown a novel biological mechanism by which brain endothelial UCP2 plays a significant role in driving the neurogenic‐to‐astrogenic fate switching of NPCs during neocortical development. Deletion of endothelial UCP2 affects the transition timing of neurogenesis to astrogenesis in NPCs/NSCs and leads to persistently increased astrocyte production at the postnatal stage. Here, the ERK1/2 pathway increased CMA1 expression to enhance AngII secretion outside brain ECs. Endotheliocyte‐driven AngII and neural precursor receptor Agtr1a also interact to activate the JAK‐STAT pathway (Figure S8D, Supporting Information). These results suggest that the brain vasculature is an important niche for NPCs/NSCs and affects the timing of stem cell fate switching during cortical development.

Blood vessels and the nervous system are important communication systems that constitute the complex brain structure.^[^ ^38^ ^]^ Blood vessels are increasingly recognized as active coordinators of tissue homeostasis and regeneration, which helps prevent unregulated disease processes.^[^ ^39^ ^]^ Therefore, vascular dysfunction affects nervous system homeostasis during brain development. We confirmed that the ERK1/2 pathway increases CMA1 expression to enhance AngII secretion outside the brain ECs. Previous studies have shown that AngII regulates adult cerebral circulation and results in significant dose‐dependent contractions in fetal middle cerebral arteries.^[^ ^40^ ^]^ The blood vessel mean diameter may be due to an increase in AngII. This finding needs to be further validated in future studies.

During cortical development, NPCs undergo self‐renewal, amplification, neurogenesis, and astrogenesis.^[^ ^41^ ^]^ The length of the neurogenic stage determines the number of neurons produced; therefore, the timing of the neurogenic‐to‐astrogenic fate switch is critical for brain development.**^[^** ^37^ **^]^** Our results demonstrated that the deletion of endothelial UCP2 did not affect the self‐renewal and expansion of neural progenitor cells. Birth dating analysis showed that cells labeled with BrdU at E14 contributed to astrocyte production but not neuronal production at E18 in endothelial UCP2 knockout mice. Our data also showed that the loss of endothelial UCP2 led to an increase in AngII by upregulating CMA1.

Previous studies have shown that the brain vasculature affects neuroglial organization, neuronal migration, and oligodendrocyte progenitor cell specification via EC‐derived secretion factors.^[^ ^12^, ^42^, ^43^ ^]^ Our data demonstrated that the EC‐derived AngII acted on AGTR1a receptor of NPCs to enhance the gp130‐JAK‐STAT signaling, which initiated gliogenesis. Previous studies have shown that JAK‐STAT signaling represses neurogenesis and simultaneously promotes gliogenesis.^[^ ^8^ ^]^ Loss of endothelial UCP2 may attenuate the capacity of neurogenic factors, resulting in a decrease in the number of neurons during the embryonic neurogenic period. Further investigation is needed to determine whether the perturbation of neurogenic to astrogenic time switch leads to neurodevelopmental disorders such as cognitive dysfunction and autism‐like behaviors. One possibility is that changes in the time and number of differentiated cells affect the establishment of the brain network and the function of neural circuits. UCP2 allows metabolic adaptation to change the availability of nutrient utilization, which is critical to the normal survival and development of hippocampal and other brain neurons.^[^ ^44^ ^]^ The key role of the brain mitochondrial adaptation is to influence neural circuits to support normal and altered adult behavior, which were supported with neuroimaging data from human neonates.^[^ ^45^ ^]^ Disturbed neurogenic‐to‐gliogenic transition leads to many neurological diseases, including de novo gliomagenesis and hydrocephalus. We further determined that the neurogenic‐to‐gliogenic switch in NPCs‐like cells is an important early stage with a burst of oncogenic alterations including the cerebrovascular specialized microenvironment. Cerebrovascular treatment at this stage was sufficient to impede gliomagenesis in vivo, which provided a proof of concept for potential early‐stage interventions against the gliomagenic trajectory.

In conclusion, our results reveal that endothelial‐derived signals on NPCs regulate the timing of the neurogenic‐to‐astrocyte fate switch, resulting in an increase in the number of astrocytes. Our study provides new insights into the regulatory link between blood vessels and sequential differentiation of NPCs.

## 4 Experimental Section

### Animals

ICR(CD‐1) pregnant mice were obtained from Vital River Laboratories. The *UCP2^flox/flox^* mice (022394‐ B6.129S*UCP2tm2.1Lowl/*J) were obtained from the Jackson Laboratory. The *Tie2‐Cre* (*TEK‐Cre*) mice were purchased from the Shanghai Model Organism. To generate *UCP2* ^ECKO^ mice, *UCP2^flox/flox^* mice were bred with *Tie2‐Cre* mice. The offsprings were confirmed by tail gene typing and PCR production sequencing. Mice were bred and maintained at 22–25 °C with a 12 h light/12 h dark cycle, and provided adequate food and water. All mice used for experiments were conducted in accordance with the guidelines of the Animal Care and Use Committee of the Institute of Zoology, Chinese Academy of Sciences (IOZ20170068).

### Plasmid Constructs

Fetal mouse brain RNA was reversed transcribed with Quant Script RT kit (Tian gen). Mouse *UCP2* cDNA was acquired by PCR and subcloned into the pCDH‐copGFP vector.

Human UCP2 shRNA1/2/3 were cloned into the pSicoR‐GFP vector. The sequences were as follows

*hUCP2‐shRNA1*: CGTGGTCAAGACGAGATACAT
*hUCP2‐shRNA2*: CGGTTACAGATCCAAGGAGAA
*hUCP2‐shRNA2*: GCCACTTCACTTCTGCCTTCG

Mouse *CMA1* cDNA was acquired by PCR and subcloned into the pCDH‐copGFP vector. *CMA1shRNA* were cloned into the pSicoR‐GFP vector. The sequences were as follows

*CMA1‐shRNA1*: CTCTGCCAACTTCAACTTTAT
*CMA1‐shRNA2*: GCAGTGGCTTCCTGATAAGAA

### Cell Culture

Human embryonic kidney 293T cells (ATCC, CRL‐1573) were cultured in Dulbecco's Modified Eagle Medium（DMEM） ,(Gibco,11995‐065) supplemented with 10% Fetal Bovine Serum（FBS） (Gibco,16000044), 1%penicillin/streptomycin (Invitrogen, 15070063). The human brain endothelial cell line, hCMEC/D3 (Millipore Sigma, SCC066) were cultured in ECM medium (Acmec Sciencell‐1001) containing 5% FBS, 10 × 10^−3^ m HEPES, and penicillin/streptomycin. The medium was changed every three days. The mouse brain endothelial cells, bEnd.3(ATCC) were cultured in DMEM (Gibco, 11995‐065) containing 10%FBS and 1%penicillin/streptomycin (Invitrogen, 15070063). The medium was changed every three days. H9 human embryonic stem cells (WiCell Research, CVCL_9773) were cultured on metrigel‐coated plates in DMEM essential 8 medium and 50 × essential 8 supplements (Gibco, A1517001). The medium was replaced with fresh medium every day.

### Isolation and Culture of Primary Neural Progenitor Cells

Primary neural progenitor cells were isolated as described previously.**^[^** ^46^ **^]^** In brief, E14 or E15 fetal brain was dissected under stereomicroscopy to remove the brainstem, cerebellum and olfactory bulb and obtain the cerebral cortex. Brain cortex was digested into cell suspension using 20 U mg^−1^ papain (Worthington, Ls003119) for 5 min at 37 °C. Then, progenitor cells were purified by washing three times with DMEM (Gibco, 11995‐065). After supernatant was removed, fresh culture medium was added to prepare cell suspension. Cell suspensions were filtered through a 70 µm strainer. Primary neural progenitor cells were cultured on Poly‐d‐Lysine (Sigma) and Laminin (Invitrogen) coated 6‐ or 24‐Well plates (costar). Cells were cultured on Poly‐d‐Lysine (Sigma, P3655) and Laminin (Invitrogen, 23017015) coated plates(costar) in proliferation medium composed of 50% DMEMF/12 medium (Gibco, 11330‐032), 50% Neurobasal medium (Gibco, 21103‐049), 50 × B27 supplement without VA (Invitrogen, 12587010), 100 ×  Gluta MAX (Invitrogen, 35050061), 10 ng mL^−1^ EGF (Invitrogen, PHG0311), 10 ng mL^−1^ bFGF (Invitrogen, PHG0026), and 100 × penicillin/streptomycin (Invitrogen). Differentiation medium composed of low glucose DMEM (Gibco, 11885‐084) supplemented with 2% FBS, 1%penicillin/streptomycin, and 50 × B27 supplement with VA (Invitrogen, 17504‐044). The medium was replaced with fresh medium every three day.

### Isolation and Culture of Primary Mouse Brain Endothelial Cells

Primary mouse brain ECs were isolated as previously described.**^[^** ^47^ **^]^** Briefly, E14 or E15 fetal brain was dissected under stereomicroscopy to remove the brainstem, cerebellum and olfactory bulb and obtain the cerebral cortex. Brain cortex was digested into cell suspension using 20 U mg^−1^ papain for 5 min at 37 °C. After filtration with 70‐µm nylon mesh, the cells were lysed with 2 mL red blood cell lysing buffer (Sigma‐Aldrich) for 2–3 min at room temperature. 10^6^–10^7^cells were incubated with anti‐CD31‐FITC (Biolegend, 102406) at 4° for 30 min. Subsequently, the labeled cells were sorted by FACSCalibur cytometer (Becton Dickinson). Finally, primary endothelial cells were cultured on collagen type I (Sigma‐Aldrich) coated 6‐ or 24‐Well plates (Costar) in EGM‐2 media (EGM‐2 BulletKit; Lonza, CC‐3162). The medium was replaced with fresh medium every three days.

### Generating Human Neural Precursor Cells

Generating human neural precursor cells (NPCs) from human embryonic stem cells procedure was performed as described previously.**^[^** ^48^ **^]^** Briefly, Human embryonic stem cells (H9) reached 70–80% in 6‐well plate, the medium was replaced with neural precursor cells differentiation medium composed of 50% DMEMF/12 medium (Gibco, 11330‐032), 50% Neurobasal medium (Gibco, 21103‐049), 100 ×  Gluta MAX (Invitrogen, 35050061), 50 × B27 supplement whit out VA (Invitrogen, 12587010), 100 × N2 supplement (Thermo Fisher, 17502048), 2 × 10^−6^ m dorsomorphin(Selleck, S7840), 3 × 10^−6^ m SB431542 (Tocris, 1614), 4 × 10^−6^ m CHIR99021 (Stemgent, 04‐0004‐10), 0.1 × 10^−6^ m compound E (EMD Chemicals, 209986‐17‐4), and 10 ng mL^−1^ human LIF (Millipore, LIF1010). After 5 d , Cells dissociated into single cells with accutase (Thermo Fisher, A1110501) were culture on metrigel‐coated 6‐ or 24‐well plates in human neural precursor cells proliferation medium composed of 50% DMEMF/12 medium (Gibco), 50%Neurobasal medium (Gibco), 100× Gluta MAX (Invitrogen), 50×B27 supplement whit out VA (Invitrogen), 100 × N2 supplement (Invitrogen), 2 × 10^−6^ m SB431542, 3 × 10^−6^ m CHIR99021, and 10 ng mL^−1^ h LIF (Millipore).

For Astrocyte‐neuronal differentiation, human neural precursor cells were culture in neuronal induction medium. The medium was composed of DMEM/F12 medium containing 50 × B27 supplement, 100 × N2 supplement, 10 ng mL^−1^ BDNF (Peprotech, 045‐02), and 10 ng mL^−1^ GDNF (Peprotech, 450‐10). 1 µg mL^−1^ Laminin (Sigma) was added after 2 d culture. LIF (10 ng mL^−1^, Millipore) or 10%FBS (Invitrogen)**^[^** ^49^ **^]^** were added after 4 d and changed the medium every two day.

### EdU Labeling

For EdU labeling, pregnant mice were injected with 50 mg kg^−1^ EdU(sigma) at E14, and the fetus were harvested at E18. Finally, the brain slices were stained with Click‐iT EdU Alexa Fluor594 Imaging Kit (Invitrogen, C10339) for further analysis.

### Immunostaining

Immunostaining procedure were performed as described previously.**^[^** ^50^ **^]^** Briefly, cultured cells were fixed in 4%PFA for 20 min at room temperature (RT), and washed three times with PBS including 0.1% Triton X‐100 (0.1%PBST). The cultured cells were blocked with 5% BSA (in 0.1%PBST) for 1 h at RT, then incubated with primary antibodies overnight at 4 °C. Brain slices fixed in 4% PFA for 30 min at RT, and washed three times with PBS containing 1% Triton X‐100 (1%PBST). The brain sections were blocked with 5% BSA (in 1%PBST) for 1 h at RT, and incubated with primary antibodies overnight at 4 °C. After secondary antibodies for 1.5 h at RT. Confocal images were captured by Carl ZeissLSM880 confocal microscope.

### Western Blotting and Co‐Immunoprecipitation

For western blotting, brain tissues or cells were lysed in RIPA lysis buffer (Solarbio) containing 1% protease inhibitor cocktail and 1% PMSF. Protein concentration was determined by Pierce BCA Protein Assay Reagent. Next, Protein samples were separated by 12% or 10% SDS–PAGE gel and transferred onto polyvinylidene fluoride (PVDF) membranes. The PVDF membranes were blocked with 5% milk or BSA in PBST (PBS containing 0.02% Tween‐20) for 1 h at room temperature, and incubated with primary antibodies at 4 °C overnight. The bands were visualized and analyzed by the Image Studio Ver 5.2 software after secondary antibodies for 1.5 h at room temperature.

For co‐immunoprecipitation, protein samples were lysed in co‐IP lysis buffer (Beyotime Biotechnology) containing 1% protease inhibitor cocktail and 1% PMSF. The supernatant was collected by centrifugation at 4 °C, and incubated with anti‐HA‐tag (MBL) or anti‐Flag‐tag magnetic beads (MBL) overnight at 4 °C. After washing three times with cold wash buffer, the bound proteins were analyzed by western blot.

### Antibodies

The following primary antibodies and dilutions were used for Immunostaining and Western blotting anti‐UCP2(Proteintech,11081‐1‐AP, Rabbit, 1:200); anti‐UCP2(Santa Cruz, sc‐6525, Goat, 1:200); anti‐UCP2 (ABclonal, A4178, Rabbit, 1:1000); anti‐biotinylated IsolectinB4(Vector Laboratories, B‐1205, 1:600); anti‐SOX2 (Cell Signaling Technology, 3728s, Rabbit, 1:1000); anti‐Tbr2(Abcam, ab23345, Rabbit, 1:1000); anti‐GFAP (Dako, Z0334, Rabbit, 1:3000); anti‐GFAP (Sigma, G6171, Mouse, 1:1000); anti‐BLBP (Abcam, ab32423, Rabbit, 1:1000); anti‐GLAST (Proteintech,20785‐1‐AP,Rabbit, 1:1000); anti‐S100*β*(Abcam, ab52642, Rabbit, 1:1000); anti‐PDGFR*β* (Abcam, ab32570, Rabbit, 1:1000); anti‐TUJ1(Sigma, T2200, Rabbit,1:1000); anti‐MAP2 (Millipore, MAB3418, Mouse, 1:500); anti‐CTIP2 (Abcam, ab18465;Rat,1:1000); anti‐SATB2 ( Abcam, ab51502, Mouse, 1:500); anti‐pCREB (Cell Signaling Technology, 9198s, Rabbit, 1:1000); Anti‐Synaptophysin (Abcam, ab8049, Rabbit, 1:2000); anti‐*β*‐Actin (Proteintech, 20536‐1‐AP, Rabbit, 1:10000); anti‐*β*‐Actin (Proteintech; 60008‐1‐Ig Mouse,1:2000); anti‐NeuN (Abcam; ab177487, Rabbit, 1:1000); anti‐IgG (Bioss; bs‐0295p, Rabbit,1:1000); anti‐ALDH1L1 (Abcam, ab56777, Rabbit, 1:2000); anti‐Flag (Sigma, F1804, Mouse, 1:3000), anti‐HA (Cell Signaling Technology, Rabbit, 1:1000); anti‐CMA1(Proteintech,18189‐1‐AP, Rabbit,1:1000); anti‐Collagen IV(Abcam; ab6586; Rabbit,1:1000); anti‐ZO‐1(Invitrogen, 40–2200,Rabbit, 1:500); anti‐Claudin 5 (Invitrogen,35‐2500, Mouse, 1:500); anti‐CD31(BD Biosciences, 553370, Rat, 1:1000); anti‐ Phospho‐p44/42 MAPK (Erk1/2) (Cell Signaling Technology, 9101S, Rabbit, 1:1000); anti‐Angiotensin II/III Antibody (Novus, NB100‐62346SS, Mouse, 1:1000).

The following secondary florescence antibodies and dilutions for Immunostaining

DAPI (2mg/ml; Sigma; D9542) was used for nuclear counterstaining; Alexa Fluor 488, Cy3, or Cy5 (Jackson ImmunoResearch, 1:1000).

The following secondary antibodies and dilutions were used for Western blotting

Donkey anti‐IgG (LI‐COR Biosciences680LT, Rabbit, 1:1000); Donkey anti‐IgG (LI‐COR Biosciences680LT, Mouse,1:1000); Donkey anti‐IgG (LI‐COR Biosciences680LT, Mouse, 1:1000); Donkey Anti‐IgG (LI‐COR Biosciences800CW, Rabbit, 1:1000); Donkey Anti‐IgG (LI‐COR Biosciences800CW, Mouse, 1:1000); Donkey anti‐IgG (LI‐COR Biosciences800LT, Rat, 1:1000).

### ELISA Assay

Brain endothelial cells were isolated from *UCP2^fl/fl^* and *UCP2^ECKO^* brain cortices and cultured in endothelial cells medium for 7 d at 37 °C. Follow manufacturer's experimental procedures, angiotensinII (AngII) concentrations were quantitatively analyzed by Mouse Angiotensin II ELISA Kit (DL‐AngII‐Mu, EVELOP). The absorbance was measured by the PowerWave XS Microplate Reader (BioTek) at 450 nm. The AngII concentration was calculated by interpolating values into a standard curve which generated from the commercially ELISA kit.

### Metabolic Assays

Primary endothelial cells were seeded in Poly‐d‐lysine‐coated XF96 cell culture microplates (Agilent Technologies, 101085‐004) at 4 × 10^4^ cells/well density in 80 µL growth media. The oxygen consumption rate (OCR) and extracellular acidification rate (ECAR) were measured using XFp extracellular flux analyzers (EFA) (Seahorse Bioscience) and XFp Cell Mito Stress Test Kit. During the assay, cells were exposed to compounds in the following order: 1.0 × 10^−3^ m oligomycin, 1.0 × 10^−3^ m FCCP, 0.5 × 10^−3^ m rotenone/antimycin A.

### RT‐PCR Analysis

The total RNA from UCP2 conditional knock‐out mouse cerebral cortex, or primary brain endothelial cells was extracted by using the Fast Quant RT Kit (Tian gen). The results of real time‐PCR were analyzed by using a SYBR qPCR master mix (Tian gen) and the ABI7500 real‐time PCR system (Applied Biosystems). *β*‐Actin expression served as an endogenous control for normalization of real‐time PCR reactions. The primers used for real‐time PCR were listed in Table S1 in the Supporting Information.

### RNA‐Sequencing Analysis

Total RNA from E15 and E18 brain endothelial cells of *UCP2^fl/lf^* and *UCP2^ECKO^* was extracted. Agilent 2100 Bioanalyze was used to quality controlled and quantified. Next, total RNA was converted to cDNA and bound the library, and RNA‐sequencing analysis was used by the Illumina HiSeq 2500 platform in Annoroad Genomics.

### Statistical Analysis

All Results were present as Mean ± SEM. No statistical methods were used to predetermine sample sizes. The sample sizes (*n*) were provided in the figures and figure legends. Vessels were analyzed using Imaris 9.0. All statistical analyses were performed using GraphPad Prism 6.0. Data normality distribution was accessed before choosing statistical tests. Statistical analysis was performed using unpaired two‐tailed Student's *t* test between two experimental groups or one‐way ANOVA with Dunnett's multiple‐comparison correction among three groups. *P < 0.05, **P < 0.01, ***P < 0.001, n.s., not significant.

## Conflict of Interest

The authors declare no conflict of interest.

## Author Contributions

W.W. and L.S. contributed equally to this work. W.W., L.S., and J.J. designed the research; W.W. performed the research, drafted the manuscript and data analyses. L.S. contributed to image analysis and interpreted the data. Y.W., C.L., and F.J. provided some advices about experiences. J.J. supervised the project and acquired the funding support.

## Supporting information

Supporting Information

## Data Availability

The data that support the findings of this study are available in the supplementary material of this article.
