# Supplementary material for: Endothelial Cells Mediated by UCP2 Control the Neurogenic‐to‐Astrogenic Neural Stem Cells Fate Switch During Brain Development
Source: Adv Sci (Weinh). 2022 Apr 30;9(18):2105208. doi: 10.1002/advs.202105208 (PMC9218656; doi:10.1002/advs.202105208)
Supplement: Supplementary file 1 — Supporting Information [file ADVS-9-2105208-s001.pdf]

Supporting Information

Endothelial cells mediated by UCP2 control the neurogenic-to-astrogenic neural stem cells fate switch during brain development

Wenwen Wang[1,2,6] Libo Su[1,3,6], Yanyan Wang [1,3], Chenxiao Li[1,3], Fen Ji[1,3], Jianwei Jiao[1,3,4,5,7*]

Affiliations:

[1]State Key Laboratory of Stem Cell and Reproductive Biology, Institute of Zoology, Chinese Academy of Sciences, Beijing, China.

[2]School of Life Sciences, University of Science and Technology of China, Hefei, China.

[3]University of Chinese Academy of Sciences, Beijing, China.

[4]Co-Innovation Center of Neuroregeneration, Nantong University, Nantong, China.

[5] Beijing Institute for Stem Cell and Regenerative Medicine, Institute for Stem Cell and Regeneration, Chinese Academy of Sciences, Beijing, China.

[6]These authors contributed equally

[7]Lead Contact

*Correspondence: jwjiao@ioz.ac.cn

Supplementary Table 1: QPCR Primers

Name	Sequence(5‘-3’)
MouseUCP2-RT-F	TGTGGTAAAGGTCCGCTTCC
MouseUCP2-RT-R	TCCCTTCCTCTCGTGCAATG
β-Actin-RT-F	GGCTGTATTCCCCTCCATCG
β-Actin-RT-R	CCAGTTGGTAACAATGCCATGT
CMA1-RT-F	AGCTAACCCTAGGTGTGGGA
CMA1-RT-R	GGCTGGCTCATTCACGTTTG
OMA1-RT-F	GCCGAAGCTGACAAAGTTGG
OMA1-RT-R	CTGGCAACTTGGGATAGCCA
POLG2-RT-F	CTCTGGAAGAAGGTGGCTGG
POLG2-RT-F	TTCTCGGAGGAGGGCATTTG
TWNK -RT-F	TGCATATCCCCTCCGGATCT
TWNK -RT-R	TTTCAGTCGTAGTCACCGGC
Dguok-RT-F	GGCTTCACCCCAGGTTTGTA
Dguok-RT-R	CTGCAGAGCTTCGAAGTGGA

FigureS1

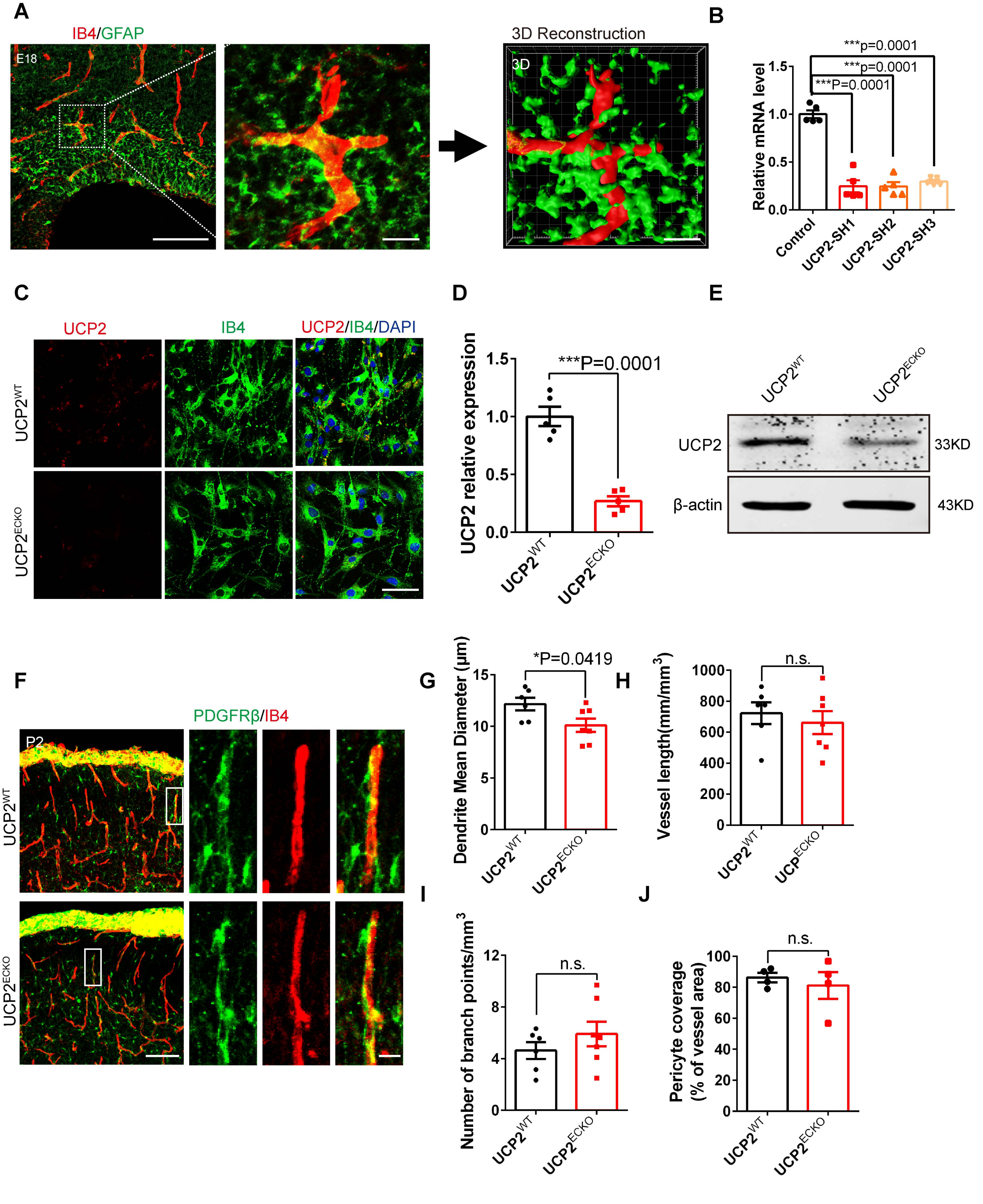
A
IB4/GFAP
E18
3D Reconstruction
3D
B
Relative mRNA level
***p=0.0001
***p=0.0001
***P=0.0001
Control
UCP2-SH1
UCP2-SH2
UCP2-SH3
C
UCP2
IB4
UCP2/IB4/DAPI
UCP2^WT
UCP2^ECKO
D
UCP2 relative expression
***P=0.0001
E
UCP2
β-actin
33KD
43KD
F
PDGFRβ/IB4
P2
G
Dendrite Mean Diameter (μm)
*P=0.0419
H
Vessel length(mm/mm^3)
n.s.
I
Number of branch points/mm^3
n.s.
J
Pericyte coverage (% of vessel area)
n.s.

Figure S1. Endothelial UCP2 deletion reduces the blood vessel diameter.

(**A**) Confocal immunofluorescence image of IB4 and GFAP at E18. The right is higher magnification images and 3D reconstructions of z-stacks of brain vessels and astrocytes. Scale bars, 100μm (left), 10μm (right).

(**B**) RT-PCR was performed to detect the mRNA levels of UCP2 in endothelial cells, NPC, neurons and astrocytes, ***P < 0.001 (mean ± SEM, unpaired two-tailed Student's *t* test, n = 3 independent experiments).

(**C**) Confocal immunofluorescence image of IB4 and UCP2 in the $UCP2^{WT}$ and $UCP2^{ECKO}$ isolated brain endothelial cells. Scale bar, 50μm.

(**D**) Quantification of UCP2 relative expression and showing the expression of UCP2 was depleted in $UCP2^{ECKO}$ brain endothelial cells.***P<0.001(mean ± SEM, unpaired two-tailed Student's *t* test, n =5 each group from 3 independent experiments).

(**E**)Western blot analysis of UCP2 expression levels in the $UCP2^{WT}$ and $UCP2^{ECKO}$ isolated brain endothelial cells. β-actin was detected as loading control.

(**F**) Confocal immunofluorescence image of IB4 and PDGFRβ in the $UCP2^{WT}$ and $UCP2^{ECKO}$ cortical sections at P2. The right magnification images showing a decreased mean diameter in $UCP2^{ECKO}$ cortical sections. Scale bars, 100μm (left),10μm (right).

(**G-I**) Quantification of the mean diameter (G), vessel length(H) and branch points(I), and showing a decreased mean diameter in $UCP2^{ECKO}$ cortical sections. *P<0.05, n.s., not significant (mean ± SEM, unpaired two-tailed Student's *t* test, $UCP2^{WT}$ n =6 mice; $UCP2^{ECKO}$ n=7 mice).

(**J**) Quantification of the PDGFRβ^{+}pericytes coverage showing no changes between

UCP2^{WT} and *UCP2*^{ECKO} mice. n.s., not significant (mean ± SEM, unpaired two-tailed Student's *t* test, n =4 mice each group).

Data are represented as means ± SEM. unpaired two-tailed Student's *t* test; At least three biological replicates are shown. n.s., not significant. *P<0.05, ***P<0.001.

FigureS2

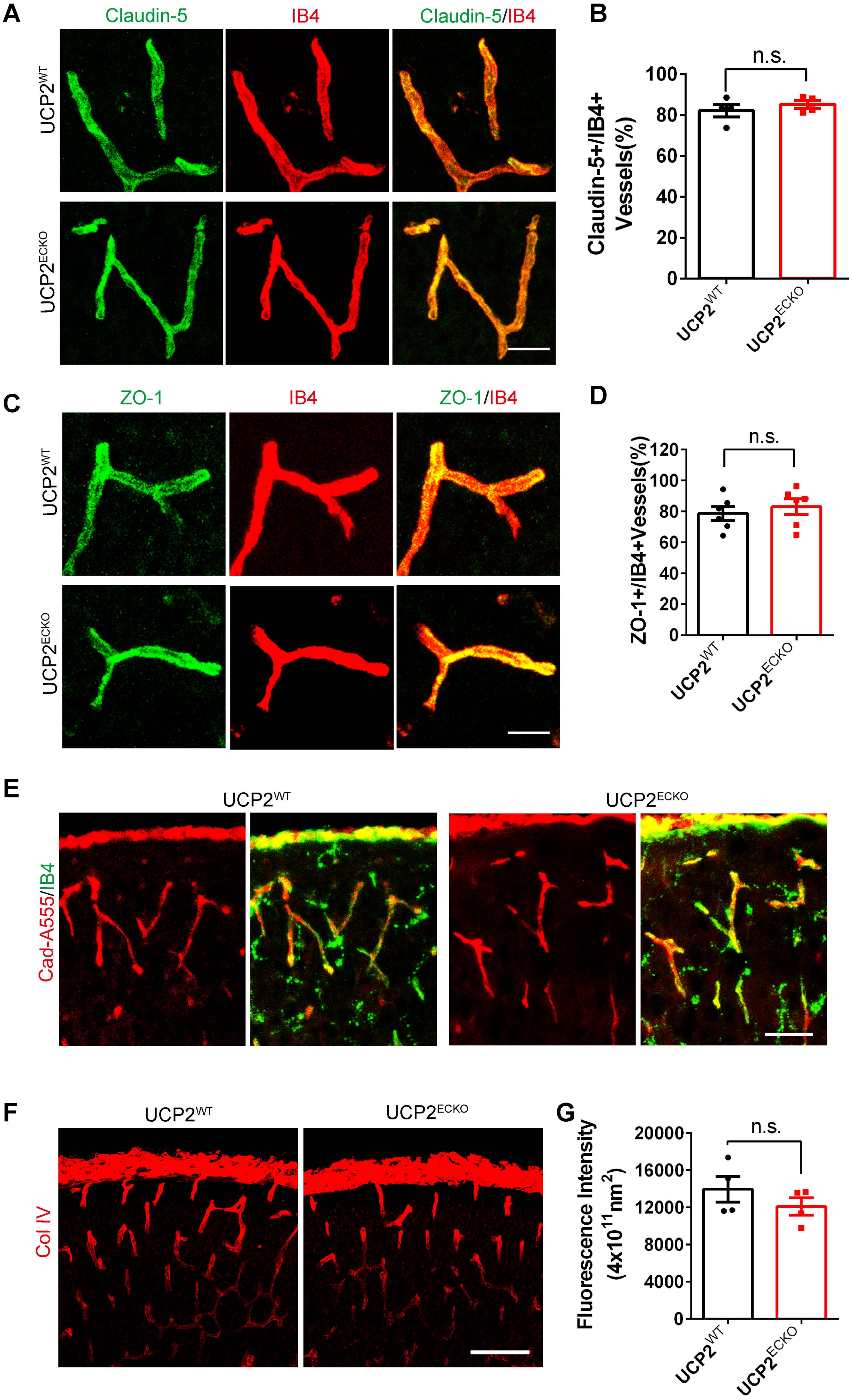

A
Claudin-5
IB4
Claudin-5/IB4
UCP2WT
UCP2ECKO
B
n.s.
Claudin-5+/IB4+ Vessels(%)
100
80
60
40
20
0
UCP2WT
UCP2ECKO
C
ZO-1
IB4
ZO-1/IB4
UCP2WT
UCP2ECKO
D
n.s.
ZO-1+/IB4+Vessels(%)
120
100
80
60
40
20
0
UCP2WT
UCP2ECKO
E
UCP2WT
UCP2ECKO
Cad-A555/IB4
F
UCP2WT
UCP2ECKO
Col IV
G
n.s.
Fluorescence Intensity (4x10^11nm^2)
20000
16000
12000
8000
4000
0
UCP2WT
UCP2ECKO

Figure S2. Endothelial UCP2 deletion does not affect blood-brain barrier integrity.

(**A**) Confocal immunofluorescence image of IB4 and Claudin-5 in the $UCP2^{WT}$ and $UCP2^{ECKO}$ mice. Scale bar, 20µm.

(**B**) Quantification of the percent of Claudin-5^{+} IB4^{+} vessels showing no statistical difference between $UCP2^{WT}$ and $UCP2^{ECKO}$ mice. n.s., not significant. (mean ± SEM, unpaired two-tailed Student's *t* test, n =4 mice each group).

(**C**) Confocal immunofluorescence image of IB4 and ZO-1 in the $UCP2^{WT}$ and $UCP2^{ECKO}$ mice. Scale bar, 20µm.

(**D**) Quantification of the percent of ZO-1^{+} IB4^{+} vessels showing no statistical difference between $UCP2^{WT}$ and $UCP2^{ECKO}$ mice. n.s., not significant. (mean ± SEM, unpaired two-tailed Student's *t* test, n =6 mice each group).

(**E**) Confocal images of IB4 and Cad-A555 showing no cadaverine extravasation in $UCP2^{WT}$ and $UCP2^{ECKO}$ brain cortex from the blood vessels. Scale bar, 100µm.

(**F**) Confocal images of Col IV showing no statistical difference between $UCP2^{WT}$ and $UCP2^{ECKO}$ brain cortex. Scale bar, 100µm.

(**G**) Quantification of the fluorescence intensity of Col IV staining showing no statistical difference between $UCP2^{WT}$ and $UCP2^{ECKO}$ mice. n.s., not significant. (mean ± SEM, unpaired two-tailed Student's *t* test, n =4 mice each group).

Data are represented as means ± SEM. unpaired two-tailed Student's *t* test; At least three biological replicates are shown. n.s., not significant. ***P<0.001.

Figure S3

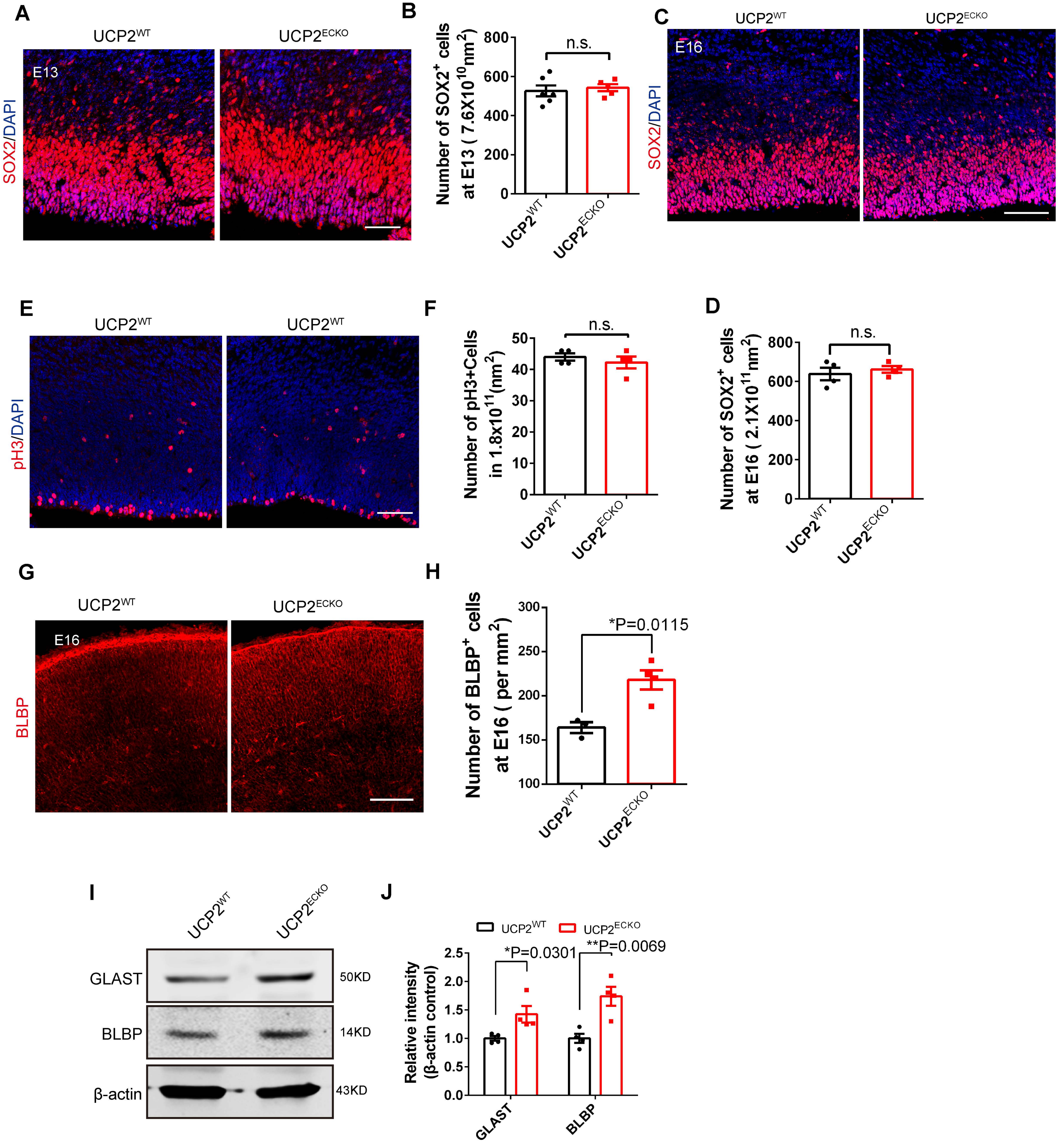
A
UCP2WT
UCP2ECKO
E13
SOX2/DAPI
B
Number of SOX2+ cells at E13 (7.6X10^10 nm^2)
n.s.
800
600
400
200
0
UCP2WT
UCP2ECKO
C
UCP2WT
UCP2ECKO
E16
SOX2/DAPI
E
UCP2WT
UCP2WT
pH3/DAPI
F
Number of pH3+Cells in 1.8x10^11(nm^2)
n.s.
50
40
30
20
10
0
UCP2WT
UCP2ECKO
D
Number of SOX2+ cells at E16 (2.1X10^11nm^2)
n.s.
800
600
400
200
0
UCP2WT
UCP2ECKO
G
UCP2WT
UCP2ECKO
E16
BLBP
H
Number of BLBP+ cells at E16 (per mm^2)
*P=0.0115
300
250
200
150
100
UCP2WT
UCP2ECKO
I
UCP2WT
UCP2ECKO
GLAST
50KD
BLBP
14KD
β-actin
43KD
J
UCP2WT
UCP2ECKO
*P=0.0301
**P=0.0069
Relative intensity (β-actin control)
2.5
2.0
1.5
1.0
0.5
0.0
GLAST
BLBP

Figure S3. Endothelial UCP2 deletion promotes astrocyte progenitor production.

(**A**) Confocal immunofluorescence image of $SOX2^{+}$ cells in E13 $UCP2^{WT}$ and $UCP2^{ECKO}$ mice. Scale bar, 50μm.

(**B**) Quantification showing that number of $SOX2^{+}$ precursor cells have comparable levels between $UCP2^{WT}$ and $UCP2^{ECKO}$ mice. n.s., not significant. (mean ± SEM, unpaired two-tailed Student's *t* test, $UCP2^{WT}$ n =6mice; $UCP2^{ECKO}$ n=5 mice).

(**C**) Confocal immunofluorescence image of $SOX2^{+}$ cells in E16 $UCP2^{WT}$ and $UCP2^{ECKO}$ mice. Scale bar, 100μm.

(**D**) Quantification showing that number of $SOX2^{+}$ precursor cells have comparable levels between $UCP2^{WT}$ and $UCP2^{ECKO}$ mice. n.s., not significant. (mean ± SEM, unpaired two-tailed Student's *t* test, n=4 mice each group).

(**E**) Confocal immunofluorescence image of $pH3^{+}$ cells in E13 $UCP2^{WT}$ and $UCP2^{ECKO}$ mice. Scale bar, 50μm.

(**F**) Quantification showing that number of $pH3^{+}$ cells had comparable levels between $UCP2^{WT}$ and $UCP2^{ECKO}$ mice. n.s., not significant. (mean ± SEM, unpaired two-tailed Student's *t* test, n=4 mice each group).

(**G**) Confocal immunofluorescence image of $BLBP^{+}$ cells in E16 $UCP2^{WT}$ and $UCP2^{ECKO}$ mice. Scale bars, 100μm.

(**H**) Quantification showing the increased number of $BLBP^{+}$ cells in $UCP2^{ECKO}$ mice. *$P<0.05$ (mean ± SEM, unpaired two-tailed Student's *t* test, $UCP2^{WT}$ n =3 mice; $UCP2^{ECKO}$ n=4 mice).

(**I**) Western blot analysis of the expression levels of astrocyte progenitor marker

GLAST and BLBP. β-actin was detected as loading control.

(**J**) Statistics of relative intensity of GLAST and BLBP showing the increased expression of $GLAST^{+}$ and $BLBP^{+}$ cells in *UCP2*^{ECKO} mice. *P<0.05, **P<0.01 (mean ± SEM, unpaired two-tailed Student's *t* test, n =4 mice each group).

Data are represented as means ± SEM. unpaired two-tailed Student's *t* test; At least three biological replicates are shown. n.s., not significant. *P<0.05, **P<0.01, ***P<0.001.

Figure S4

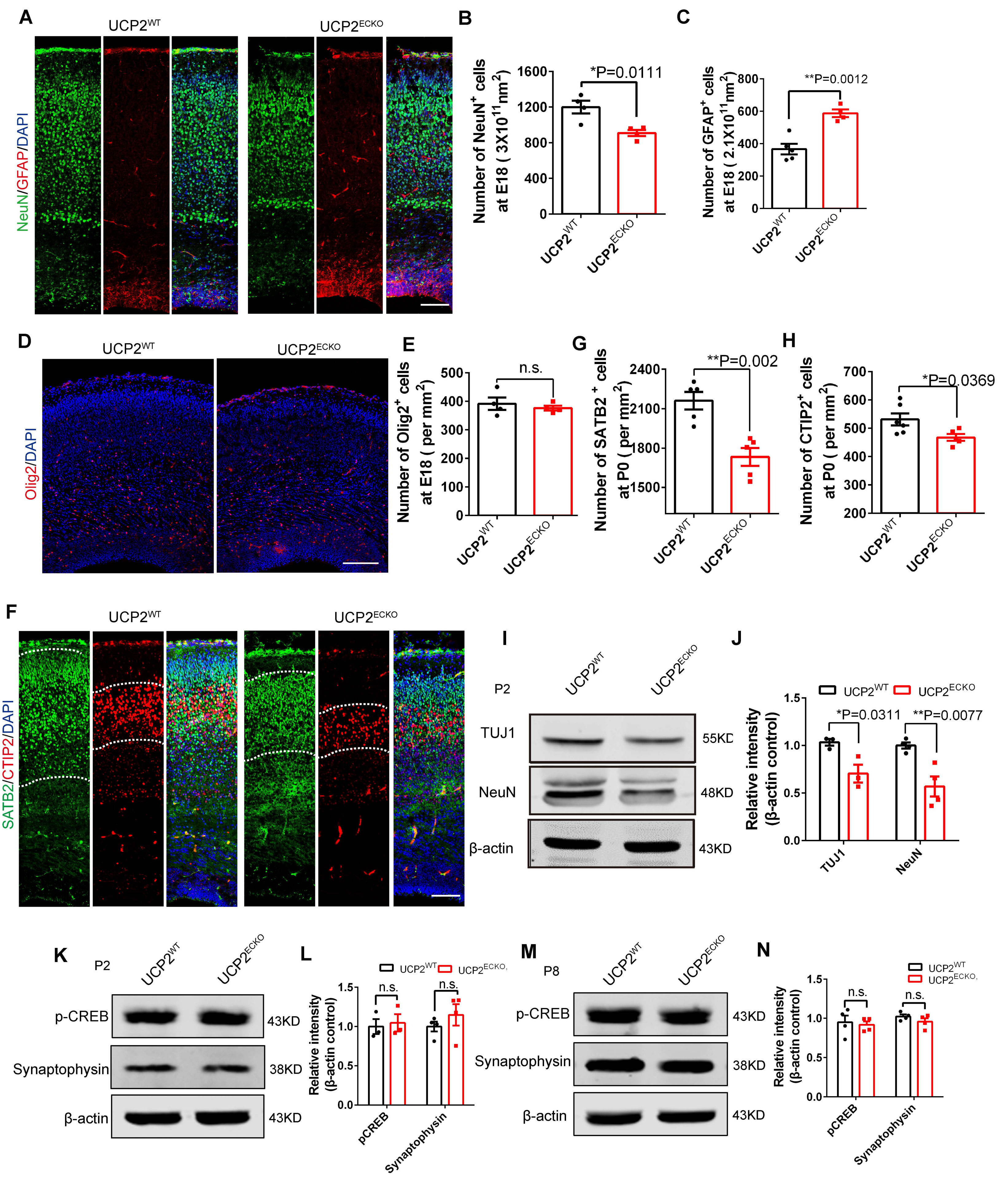
A
UCP2WT
UCP2ECKO
NeuN/GFAP/DAPI
B
Number of NeuN+ cells at E18 (3X10^11 nm^2)
*P=0.0111
C
Number of GFAP+ cells at E18 (2.1X10^11 nm^2)
**P=0.0012
D
Olig2/DAPI
E
Number of Olig2+ cells at E18 (per mm^2)
n.s.
G
Number of SATB2+ cells at P0 (per mm^2)
**P=0.002
H
Number of CTIP2+ cells at P0 (per mm^2)
*P=0.0369
F
SATB2/CTIP2/DAPI
I
P2
TUJ1
NeuN
β-actin
55KD
48KD
43KD
J
Relative intensity (β-actin control)
*P=0.0311
**P=0.0077
K
P2
p-CREB
Synaptophysin
β-actin
43KD
38KD
43KD
L
n.s.
pCREB
Synaptophysin
M
P8
p-CREB
Synaptophysin
β-actin
43KD
38KD
43KD
N
n.s.
pCREB
Synaptophysin

Figure S4. Endothelial UCP2 deletion affects neuronal development.

(**A**) Confocal immunofluorescence image of $NEUN^+$ and $GFAP^+$ cells in E18 $UCP2^{WT}$ and $UCP2^{ECKO}$ mice. Scale bar, 100μm.

(**B**) Quantification showing decreased number of $NEUN^+$ cells in $UCP2^{ECKO}$ mice. *P<0.05 (mean ± SEM, unpaired two-tailed Student's *t* test, n=4 mice each group).

(**C**) Quantification showing increased number of $GFAP^+$ cells in $UCP2^{ECKO}$ mice. **P<0.01 (mean ± SEM, unpaired two-tailed Student's *t* test, $UCP2^{WT}$ n =5 mice; $UCP2^{ECKO}$ n=4 mice).

(**D**) Confocal immunofluorescence image of $Olig2^+$ cells in E18 $UCP2^{WT}$ and $UCP2^{ECKO}$ mice. Scale bar, 100μm.

(**E**) Quantification of the number of $Olig2^+$ cells showing no statistical difference between $UCP2^{WT}$ and $UCP2^{ECKO}$ mice. n.s., not significant. (mean ± SEM, unpaired two-tailed Student's *t* test, n =4 independent experiments).

(**F**) Confocal immunofluorescence image of $SATB2^+$ and $CTIP2^+$ cells in E18 $UCP2^{WT}$ and $UCP2^{ECKO}$ mice. Scale bar, 100μm.

(**G**) Quantification showing decreased number of $SATB2^+$ cells in $UCP2^{ECKO}$ mice. **P<0.01 (mean ± SEM, unpaired two-tailed Student's *t* test, n=5 mice each group).

(**H**) Quantification showing decreased number of $CTIP2^+$ cells in $UCP2^{ECKO}$ mice. **P<0.05 (mean ± SEM, unpaired two-tailed Student's *t* test, $UCP2^{WT}$ n =6 mice; $UCP2^{ECKO}$ n=5 mice).

(**I**) Western blot analysis of the expression levels of neuron marker TUJ1and NEUN. β-actin was detected as loading control.

(**J**) Statistics of relative intensity of TUJ1and NEUN showing decreased the expression of $TUJ1^+$ and $NEUN^+$ cells in *UCP2*^{ECKO} mice. *P<0.05, **P<0.01 (mean ± SEM, unpaired two-tailed Student's *t* test, TUJ1 n =4 independent experiments; NEUN n =4 independent experiments).

(**K**) Western blot analysis of the expression levels of synaptic marker p-CREB and synaptophysin at P2. β-actin was detected as loading control.

(**L**) Statistics of relative intensity of p-CREB and synaptophysin. n.s., not significant. (mean ± SEM, unpaired two-tailed Student's *t* test, p-CREB n =3 mice each group; NEUN n =4 mice each group).

(**M**) Western blot analysis of the expression levels of synaptic marker p-CREB and synaptophysin at P8. β-actin was detected as loading control.

(**N**) Statistics of relative intensity of p-CREB and synaptophysin. n.s., not significant. (mean ± SEM, unpaired two-tailed Student's *t* test, p-CREB n =4 mice each group; NEUN n =4 mice each group).

Data are represented as means ± SEM. At least three biological replicates are shown. unpaired two-tailed Student's *t* test; n.s., not significant. *P<0.05, **P<0.01.

Figure S5

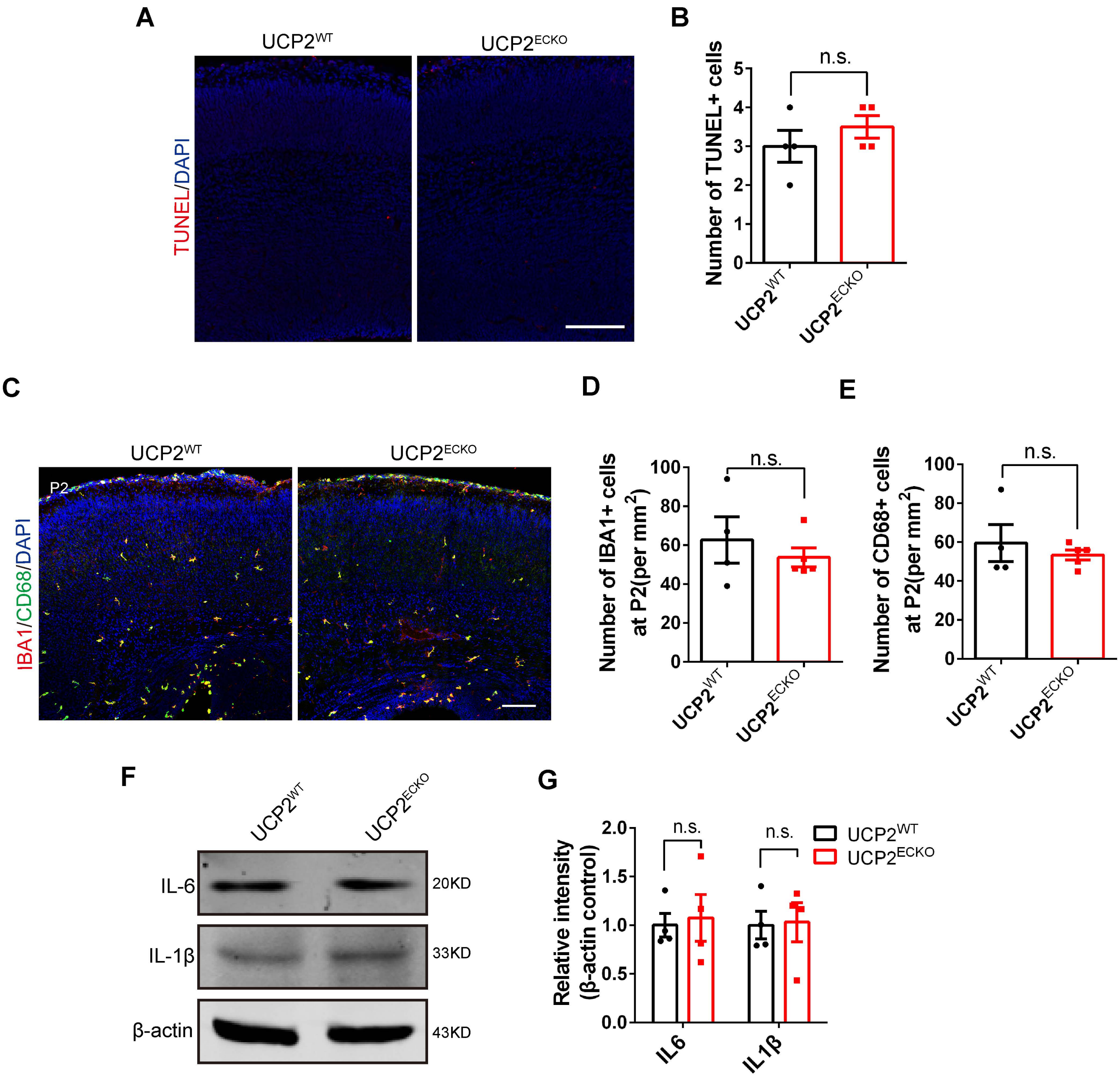

A
UCP2WT
UCP2ECKO
TUNEL/DAPI
B
n.s.
Number of TUNEL+ cells
UCP2WT
UCP2ECKO
C
UCP2WT
UCP2ECKO
P2
IBA1/CD68/DAPI
D
n.s.
Number of IBA1+ cells at P2(per mm2)
UCP2WT
UCP2ECKO
E
n.s.
Number of CD68+ cells at P2(per mm2)
UCP2WT
UCP2ECKO
F
UCP2WT
UCP2ECKO
IL-6
20KD
IL-1β
33KD
β-actin
43KD
G
n.s.
n.s.
UCP2WT
UCP2ECKO
Relative intensity (β-actin control)
IL6
IL1β

Figure S5. Endothelial UCP2 deletion does not affect inflammatory response.

(**A**) Confocal immunofluorescence image of $TUNEL^+$ cells in *UCP2*^{WT} and *UCP2*^{ECKO} mice. Scale bar, 100μm.

(**B**) Quantification of the number of $TUNEL^+$ cells showing no statistical difference between *UCP2*^{WT} and *UCP2*^{ECKO} mice. n.s., not significant. (mean ± SEM, unpaired two-tailed Student's *t* test, n =4 mice each group).

(**C**) Confocal immunofluorescence image of $IBA1^+$ and $CD68^+$ cells in *UCP2*^{WT} and *UCP2*^{ECKO} mice. Scale bar, 100μm.

(**D** and **E**) Quantification of the number of $IBA1^+$ and $CD68^+$ cells showing no statistical difference between *UCP2*^{WT} and *UCP2*^{ECKO} mice. n.s., not significant. (mean ± SEM, unpaired two-tailed Student's *t* test, *UCP2*^{WT} n =4 mice; *UCP2*^{ECKO} n =5 mice).

(**F**) Western blot analysis of the expression levels of IL6 and IL1β. β-actin was detected as loading control.

(**G**) Statistics of relative intensity of IL6 and IL1β showing no statistical difference between *UCP2*^{WT} and *UCP2*^{ECKO} mice. n.s., not significant. (mean ± SEM, unpaired two-tailed Student's *t* test, n =4 mice each group).

Data are represented as means ± SEM. unpaired two-tailed Student's *t* test; n.s., not significant. n.s., not significant.

FigureS6

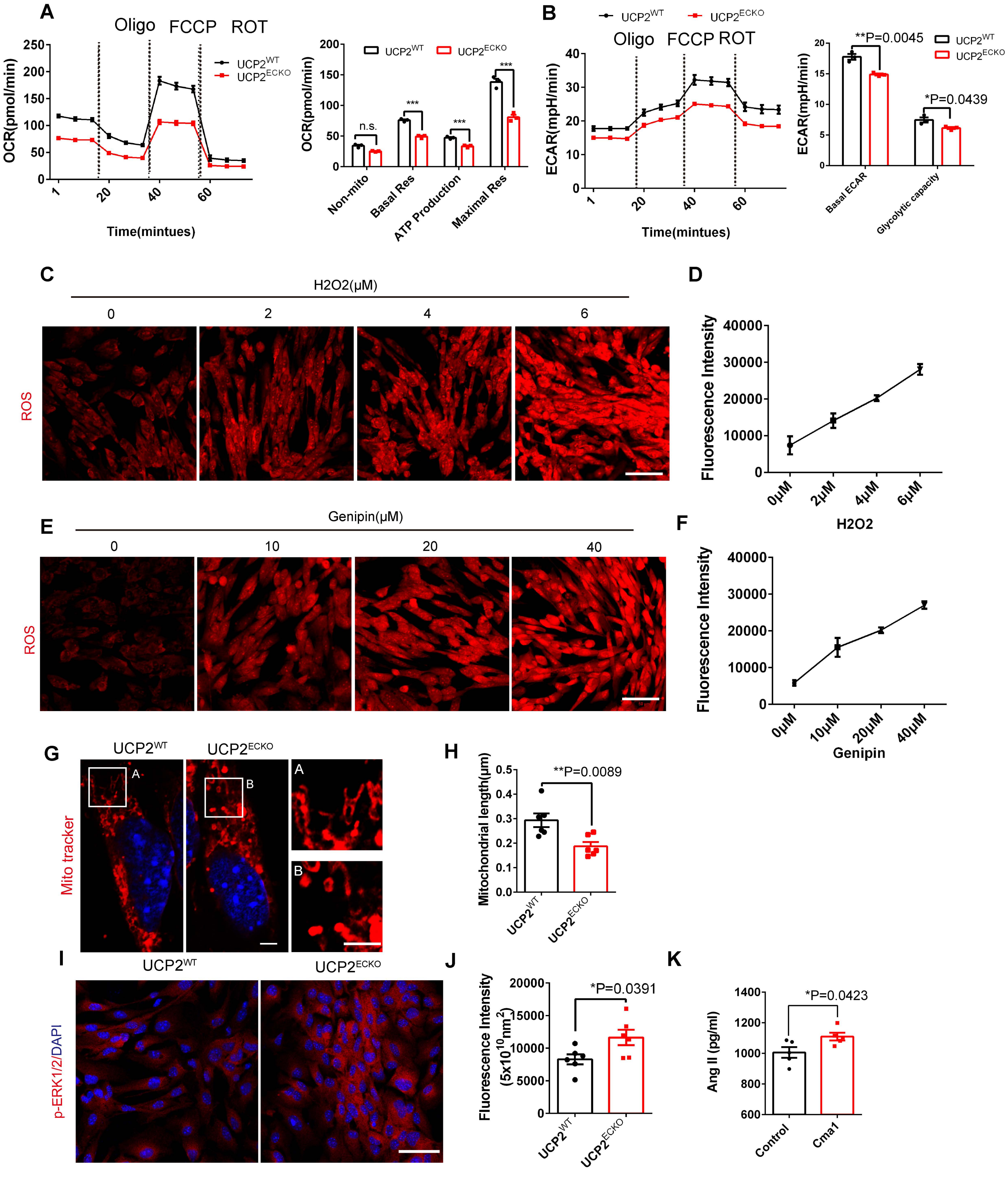

A
Oligo FCCP ROT
OCR(pmol/min)
UCP2WT
UCP2ECKO
Time(mintues)
n.s.
***
Non-mito
Basal Res
ATP Production
Maximal Res
B
ECAR(mpH/min)
**P=0.0045
*P=0.0439
Basal ECAR
Glycolytic capacity
C
H2O2(μM)
ROS
D
Fluorescence Intensity
0μM
2μM
4μM
6μM
E
Genipin(μM)
F
H2O2
10μM
20μM
40μM
Genipin
G
Mito tracker
H
Mitochondrial length(μm)
**P=0.0089
I
p-ERK1/2/DAPI
J
Fluorescence Intensity (5x10^10 nm^2)
*P=0.0391
K
Ang II (pg/ml)
*P=0.0423
Control
Cma1

Figure S6. Deletion of UCP2 in endotheliocyte upregulates ERK1/2 signaling by ROS.

(**A**) Graphs showing oxygen consumption rate (OCR) in primary endothelial cells of *UCP2*^{WT} and *UCP2*^{ECKO}. n.s., not significant. ***P<0.001 (mean ± SEM, unpaired two-tailed Student's *t* test, n = 3 mice each group, Basal Res P=0.0002; ATP Production P=0.0009; Maximal Res P=0.001).

(**B**) Graphs showing extracellular acidification rate (EACR) in primary endothelial cells of *UCP2*^{WT} and *UCP2*^{ECKO}. *P<0.05. (mean ± SEM, unpaired two-tailed Student's *t* test, n = 3 mice each group).

(**C**) Confocal immunofluorescence image of ROS levels in primary endothelial cells treated with different concentration hydrogen peroxide (H_2O_2). Scale bar, 50µm.

(**D**) Quantification of the fluorescence intensity of ROS showing that ROS production also tends to increase with the increasing concentration of H_2O_2. (mean ± SEM, n = 3 independent experiments).

(**E**) Confocal immunofluorescence image of ROS levels in primary endothelial cells treated with different concentration UCP2 selective inhibitor genipin. Scale bar, 50µm.

(**F**) Quantification of the fluorescence intensity of ROS showing that ROS production also tends to increase with the increasing concentration of genipin. (mean ± SEM, n = 3 independent experiments).

(**G**) Representative images of EC mitochondrial morphology of *UCP2*^{WT} and *UCP2*^{ECKO} mice by Mito tracker red staining. A and B are higher magnification images. Scale bar, 5µm.

(**H**) Graphs showing mitochondrial length in EC. P=0.0089 (mean ± SEM, unpaired two-tailed Student's *t* test, n = 6 from 3 independent experiments).

(**I**) Confocal immunofluorescence image of p-ERK1/2 in primary endothelial cells from $UCP2^{WT}$ and $UCP2^{ECKO}$mice.

(**J**) Quantification of the fluorescence intensity of p-ERK1/2 showing increased p-ERK1/2 cells in in primary endothelial cells from $UCP2^{WT}$ and $UCP2^{ECKO}$ mice. *P<0.05 (mean ± SEM, unpaired two-tailed Student's *t* test, n =6 from 3 independent experiments).

(**K**) ELISA analysis by collecting supernatant in primary endothelial cells infected with control and CMA1 showing increased Ang II in primary endothelial cells infected with CMA1. *P<0.05 (mean ± SEM, unpaired two-tailed Student's *t* test, n =5 each group from 3 independent experiments).

Data are represented as means ± SEM. unpaired two-tailed Student's *t* test; At least three biological replicates are shown. n.s., not significant. *P<0.05, **P<0.01, ***P<0.001.

Figure S7

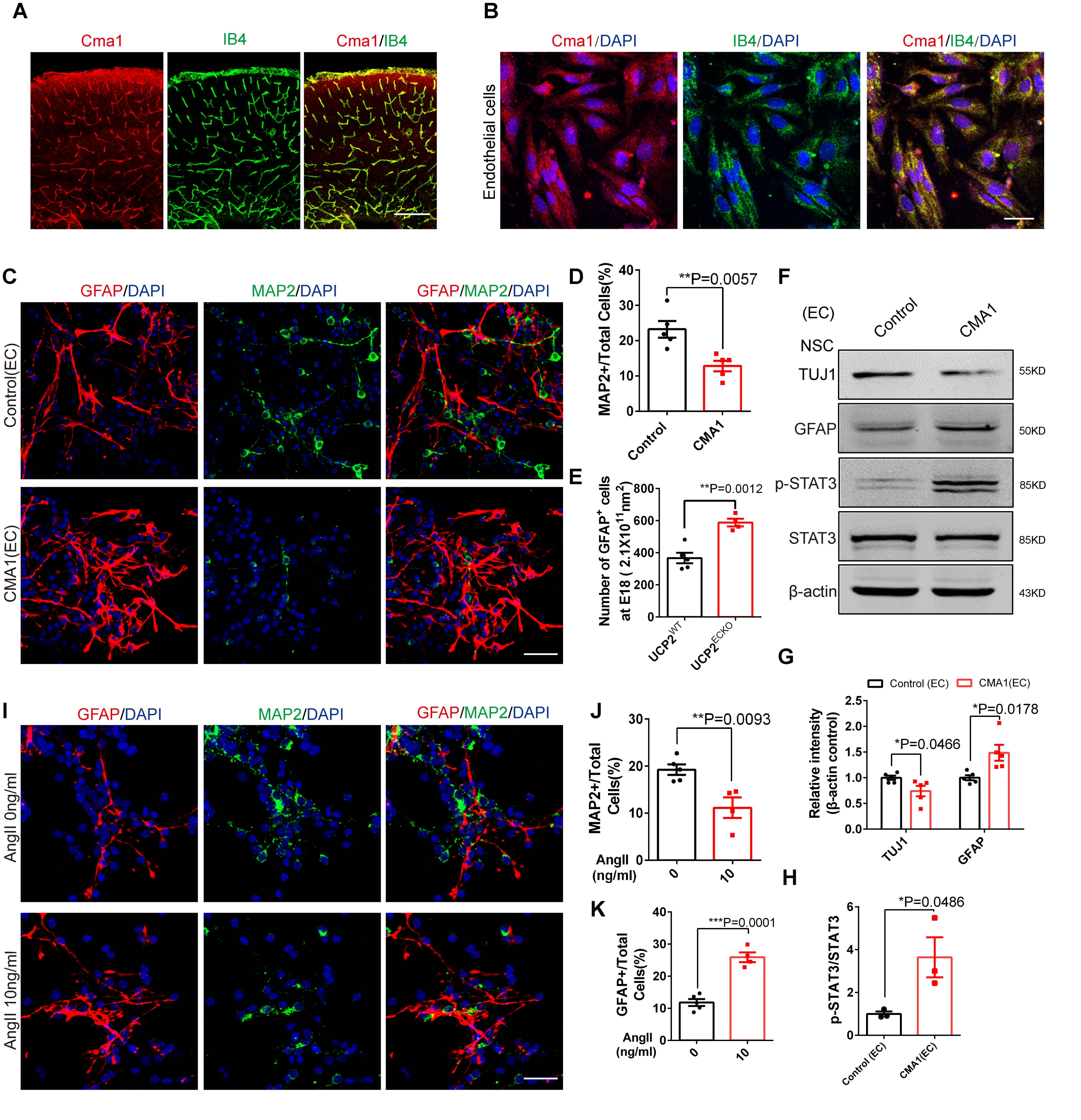
A
Cma1
IB4
Cma1/IB4
B
Cma1/DAPI
IB4/DAPI
Cma1/IB4/DAPI
Endothelial cells
C
GFAP/DAPI
MAP2/DAPI
GFAP/MAP2/DAPI
Control(EC)
CMA1(EC)
D
**P=0.0057
MAP2+/Total Cells(%)
Control
CMA1
E
**P=0.0012
Number of GFAP+ cells at E18 (2.1X10^11 nm^2)
UCP2^WT
UCP2^ECKO
F
(EC)
Control
CMA1
NSC
TUJ1
55KD
GFAP
50KD
p-STAT3
85KD
STAT3
85KD
β-actin
43KD
G
Control (EC)
CMA1(EC)
*P=0.0466
*P=0.0178
Relative intensity (β-actin control)
TUJ1
GFAP
H
*P=0.0486
p-STAT3/STAT3
Control (EC)
CMA1(EC)
I
GFAP/DAPI
MAP2/DAPI
GFAP/MAP2/DAPI
AngII 0ng/ml
AngII 10ng/ml
J
**P=0.0093
MAP2+/Total Cells(%)
AngII (ng/ml)
K
***P=0.0001
GFAP+/Total Cells(%)
AngII (ng/ml)

Figure S7. CMA1 is downstream target of UCP2.

(**A**) Confocal immunofluorescence image of CMA1 and IB4 showing that CMA1 was expressed in IB4 -labeled brain vessels. Scale bar, 100μm.

(**B**) Confocal immunofluorescence image of CMA1 and IB4 showing that CMA1 was expressed in IB4 -labeled brain endothelial cells. Scale bar, 50μm.

(**C**) Confocal immunofluorescence image of $GFAP^+$ and $MAP2^+$ cells of primary neural precursor cells co-cultured with endothelial cells infected with CMA1 and control. Scale bar, 50μm.

(**D** and **E**) Quantification of the percent of $MAP2^+$ neurons and $GFAP^+$ astrocytes showing decreased $MAP2^+$ neurons and increased $GFAP^+$ astrocytes in primary neural precursor cells co-cultured with endothelial cells infected with CMA1. **P<0.01 (mean ± SEM, unpaired two-tailed Student's *t* test, n =5 each group from 3 independent experiments).

(**F**) Western blot analysis of the expression levels of TUJ1, GFAP, p-STAT3 and STAT3. β-actin was detected as loading control.

(**G**) Statistics of relative intensity of TUJ1and GFAP showing decreased the expression of $TUJ1^+$ and the increased expression $GFAP^+$ cells in primary neural precursor cells co-cultured with endothelial cells infected with CMA1. *P<0.05 (mean ± SEM, unpaired two-tailed Student's *t* test, n =3 independent experiments).

(**H**) Statistics showing the ratio of p-STAT3 and STAT3 in primary neural precursor cells co-cultured with endothelial cells infected with CMA1. *P<0.05 (mean ± SEM, unpaired two-tailed Student's *t* test, n =3 independent experiments).

(**I**) Confocal immunofluorescence images of GFAP and MAP2 of neural precursor cells after H_2O or Ang II treatments. Scale bars, 50μm.

(**J** and **K**) Quantification of the percent of $MAP2^+$ neurons and $GFAP^+$ astrocytes showing decreased $MAP2^+$ neurons and increased $GFAP^+$ astrocytes in primary neural precursor cells after H_2O or Ang II treatments. **P<0.01, ***P<0.001 (mean ± SEM, unpaired two-tailed Student's *t* test, control n =5, Ang II n=4 from 3 independent experiments).

Data are represented as means ± SEM. unpaired two-tailed Student's *t* test; At least three biological replicates are shown. *P<0.05, **P<0.01, ***P<0.001.

Figure S8

A

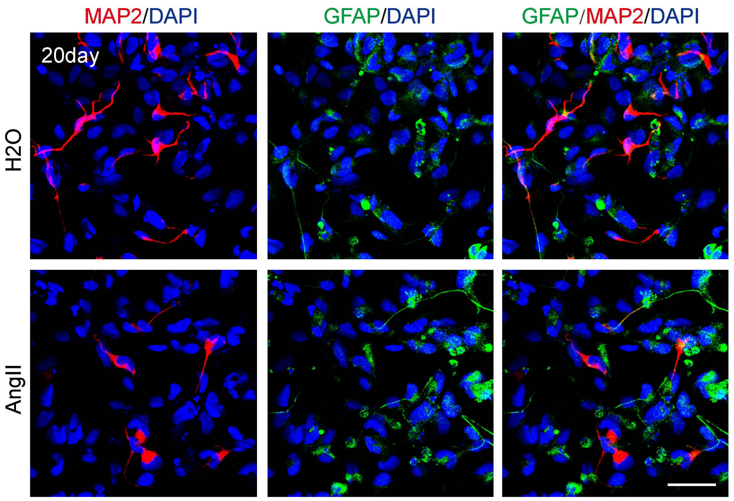

B

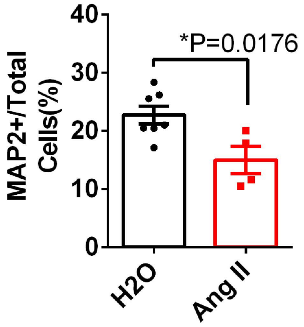

C

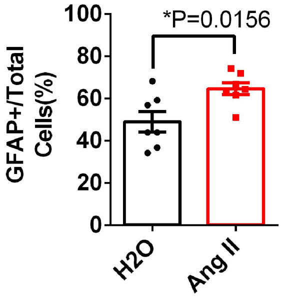

D

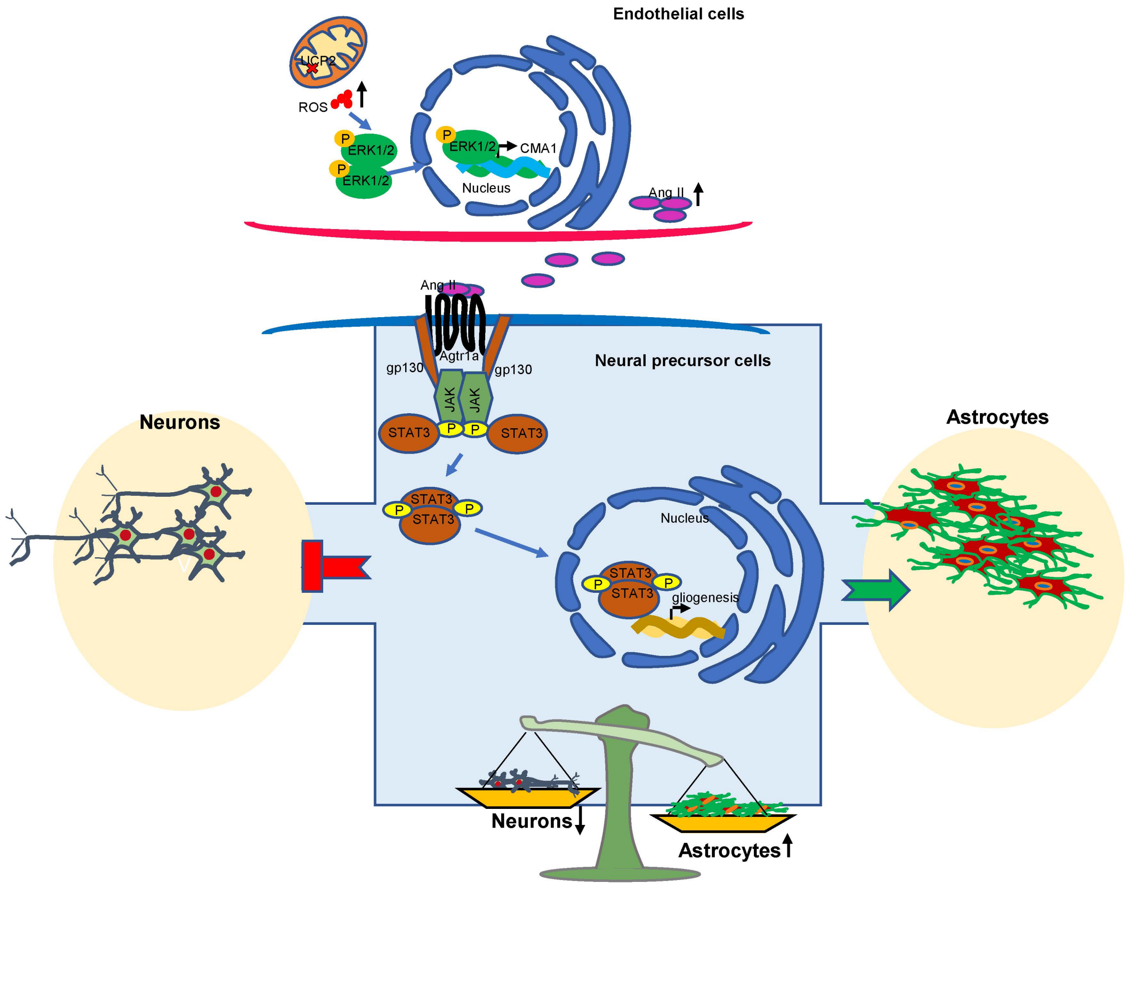

Figure S8. Ang II accelerates human NPCs differentiation toward astrocytes.

(**A**) Confocal immunofluorescence images of MAP2 and GFAP of human NPCs after H_2O or Ang II treatments. Scale bar, 50μm.

(**B**) Quantification of the percent of $MAP2^+$ neurons showing decreased $MAP2^+$ neurons s in human NPCs after H_2O or Ang II treatments. *P<0.05 (mean ± SEM, unpaired two-tailed Student's *t* test, control n =7, Ang II n=4 from 3 independent experiments).

(**C**) Quantification of the percent of $GFAP^+$ astrocytes showing increased $GFAP^+$ astrocytes in human NPCs after H_2O or Ang II treatments. *P<0.05 (mean ± SEM, unpaired two-tailed Student's *t* test, n =7 each group from 3 independent experiments).

(**D**) Model showing how endothelial UCP2 regulates the neurogenic-to-astrogenic fate switch through Ang II in the developing cortex.

Data are represented as means ± SEM. unpaired two-tailed Student's *t* test; At least three biological replicates are shown. *P<0.05.
